# Population and subspecies diversity at mouse centromere satellites

**DOI:** 10.1186/s12864-021-07591-5

**Published:** 2021-04-17

**Authors:** Uma P. Arora, Caleigh Charlebois, Raman Akinyanju Lawal, Beth L. Dumont

**Affiliations:** 1 The Jackson Laboratory, 600 Main Street, Bar Harbor, ME 04609 USA; 2 Tufts University, Graduate School of Biomedical Sciences, 136 Harrison Ave, Boston, MA 02111 USA

**Keywords:** Centromere, CENP-A, Inbred mice, Wild mice, Evolution, Bioinformatics, Satellite DNA, Genetic diversity, Mammalian genomics, *k*-mer

## Abstract

**Background:**

Mammalian centromeres are satellite-rich chromatin domains that execute conserved roles in kinetochore assembly and chromosome segregation. Centromere satellites evolve rapidly between species, but little is known about population-level diversity across these loci.

**Results:**

We developed a *k*-mer based method to quantify centromere copy number and sequence variation from whole genome sequencing data. We applied this method to diverse inbred and wild house mouse (*Mus musculus*) genomes to profile diversity across the core centromere (minor) satellite and the pericentromeric (major) satellite repeat. We show that minor satellite copy number varies more than 10-fold among inbred mouse strains, whereas major satellite copy numbers span a 3-fold range. In contrast to widely held assumptions about the homogeneity of mouse centromere repeats, we uncover marked satellite sequence heterogeneity within single genomes, with diversity levels across the minor satellite exceeding those at the major satellite. Analyses in wild-caught mice implicate subspecies and population origin as significant determinants of variation in satellite copy number and satellite heterogeneity. Intriguingly, we also find that wild-caught mice harbor dramatically reduced minor satellite copy number and elevated satellite sequence heterogeneity compared to inbred strains, suggesting that inbreeding may reshape centromere architecture in pronounced ways.

**Conclusion:**

Taken together, our results highlight the power of *k*-mer based approaches for probing variation across repetitive regions, provide an initial portrait of centromere variation across *Mus musculus,* and lay the groundwork for future functional studies on the consequences of natural genetic variation at these essential chromatin domains.

**Supplementary Information:**

The online version contains supplementary material available at 10.1186/s12864-021-07591-5.

## Background

Centromeres are chromatin domains that are essential for chromosome segregation and the maintenance of genome stability [1–4]. Centromeres serve as focal points for the assembly of the kinetochore complex, which provides the protein interface linking chromosomes to microtubules during mitosis and meiosis [1–4]. Mutations that abolish or reduce centromere function can impair kinetochore assembly and lead to spontaneous chromosome loss, cell cycle arrest, or chromosome mis-segregation [5]. Thus, the loss of centromere integrity can have adverse consequences for genome stability and represents an important mechanism leading to both cancer and infertility [6–10].

In most vertebrate species, centromeric DNA is comprised of tandem arrays of one or more satellite repeat units [11–13]. As a consequence of this satellite-rich architecture, centromeres are predisposed to high rates of structural mutation via replication slippage, unequal exchange, and transposition [9]. These processes actively contribute to satellite repeat size and sequence variability between species [14–17]. For example, in mammals, centromere repeat sizes range from 6 bp in the Chinese hamster (*Cricetulus griseus*) to 1419 bp in cattle (*Bos taurus taurus*), with GC content ranging from 28 to 74% [18]. The remarkable size and sequence variability of centromeres, combined with their critical and highly conserved cellular roles in chromosome segregation and genome stability, impose an enduring biological paradox.

Due to their inherent repeat-rich nature, centromeres persist as gaps on most reference genome assemblies. To date, only a handful of mammalian centromeres have been fully sequenced and assembled [19–21]. The near absence of high-quality reference sequences and the challenge of uniquely anchoring short reads within repeat-rich regions pose significant barriers to the discovery and analysis of genetic variation across these functionally critical regions. Consequently, the scope of centromere structural and sequence diversity within and between populations remains largely unknown.

Defining levels of centromere diversity represents a crucial first step towards understanding the potential phenotypic consequences of variation at these loci. Prior studies in humans have identified centromere variants that associate with differences in the stability of kinetochore protein binding, which can, in turn, influence the fidelity of chromosome segregation [6]. Investigations in mice and monkeyflowers (*Mimulus*) have shown that differences in centromere size can lead to biased, non-Mendelian chromosome transmission in heterozygotes, a phenomenon known as centromere drive [22–24]. However, owing to an incomplete catalog of centromere diversity and the omission of variants in these regions from GWAS and linkage studies [6, 25], the contribution of centromere variation to phenotypic variation, including disease, has yet to be fully realized.

House mice (genus *Mus*) provide an ideal system for ascertaining population level centromere satellite diversity and evaluating its functional consequences for several reasons. First, prior investigations have identified the focal *Mus musculus* centromere satellite repeat sequences and defined core features of house mouse centromere architecture [26–29]. Specifically, the *M. musculus* centromere is composed of two primary satellite domains. The minor satellite domain is a tandem array of a 120-bp sequence that cumulatively extends over ~ 1 Mb of sequence per chromosome. This satellite array delimits the region where the centromere-specific histone variant CENP-A is bound and defines the core centromere [1]. The minor satellite region is flanked by a 234-bp major satellite repeat array that extends over ~ 2 Mb of sequence per chromosome. The major satellite region forms the pericentromeric heterochromatin, which is important for sister chromatid cohesion during cell division [1, 30]. Second, mouse centromere satellite arrays are reported to be homogenous both within and between chromosomes [26, 29], a feature that simplifies the task of quantifying centromere satellite variation in genomes. This contrasts with the architecture of human centromeres, which are composed of distinct repeat arrays that form higher order repeats that vary between chromosomes [15, 16, 26]. Third, whole-genome sequences from diverse wild and inbred *M. musculus*, as well as more divergent *Mus* taxa are publicly available [31–33]. These resources enable surveys of centromere diversity along several dimensions, including among inbred strains, within natural populations, between subspecies, and between species. Finally, as the premiere mammalian biomedical model system, house mice are equipped with experimental tools and detailed phenotype catalogs that can be leveraged to test the functional consequences of centromere variation.

Here, we harness these strengths of the *M. musculus* model system to carry out the first sequence-based analysis of centromere diversity and evolution in mice. We couple *k*-mer based bioinformatic methods with experimental approaches to uncover remarkable variation in the size and sequence composition of centromeres across a panel of diverse inbred strains and wild-caught house mice. Overall, our study yields a portrait of centromere satellite diversity across a group of closely related mammals and lays the groundwork for future functional studies on the consequences of natural genetic variation at these essential chromatin domains.

## Results

### *k*-mer analysis reveals striking differences in the abundance of centromere satellite repeats across *Mus*

**Fig. 1:**
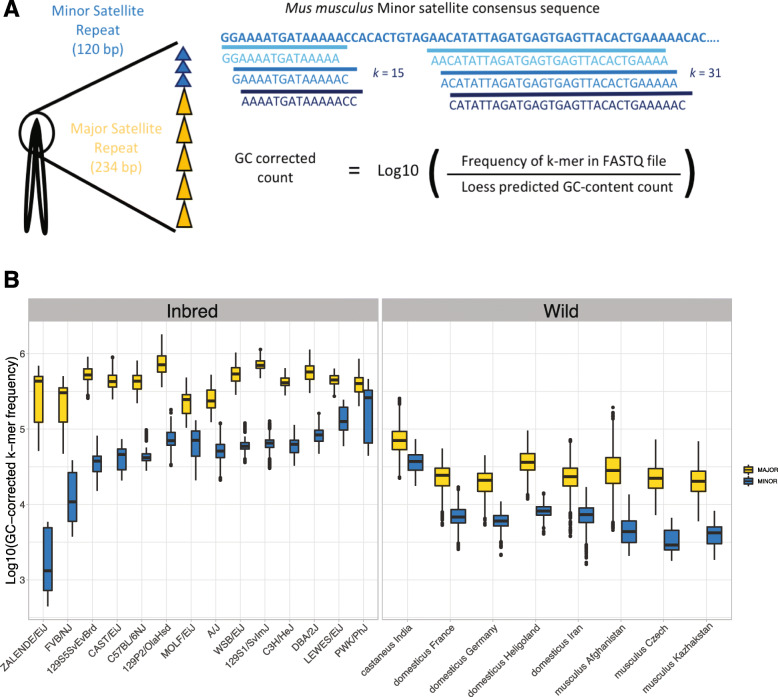
Significant differences in consensus centromere satellite copy number across *Mus musculus.* **a** Schematic overview of the approach used to quantify the frequencies of *k*-mers in centromere satellite repeats. **b** Boxplots showing the distribution of major (yellow) and minor (blue) satellite consensus 31-mer frequencies across inbred strains and wild-caught mouse populations

Standard approaches for surveying sequence diversity are not readily extendable to the centromere due to its repeat-rich architecture and gapped status on the mouse reference (mm10) assembly. To circumvent these challenges, we employed a *k*-mer based approach to quantify the diversity of satellite DNA in mouse genomes. Our *k*-mer strategy is predicated on the insight that the relative frequency of a given nucleotide word of length *k*, or *k*-mer, in a shot-gun sequencing library is proportional to its frequency in the parent sample genome. Thus, the observed frequency of a particular *k*-mer within a pool of sequenced reads can be used as a proxy for its relative abundance in a genome. Here, we focus on two values for *k*: *k* = {15, 31}. Both values yield *k*-mers with high specificity and, importantly, yield qualitatively similar results (Fig. 1, Supplementary Figure 1). We normalized *k*-mer counts to adjust for potential GC-biases introduced during library preparation and confirmed through rigorous comparisons of replicate libraries for individual samples that our corrected *k*-mer counts provided a reliable readout of the relative frequency of nucleotide motifs in diverse mouse genomes (Supplementary Figure 2; See Methods).

**Fig. 2:**
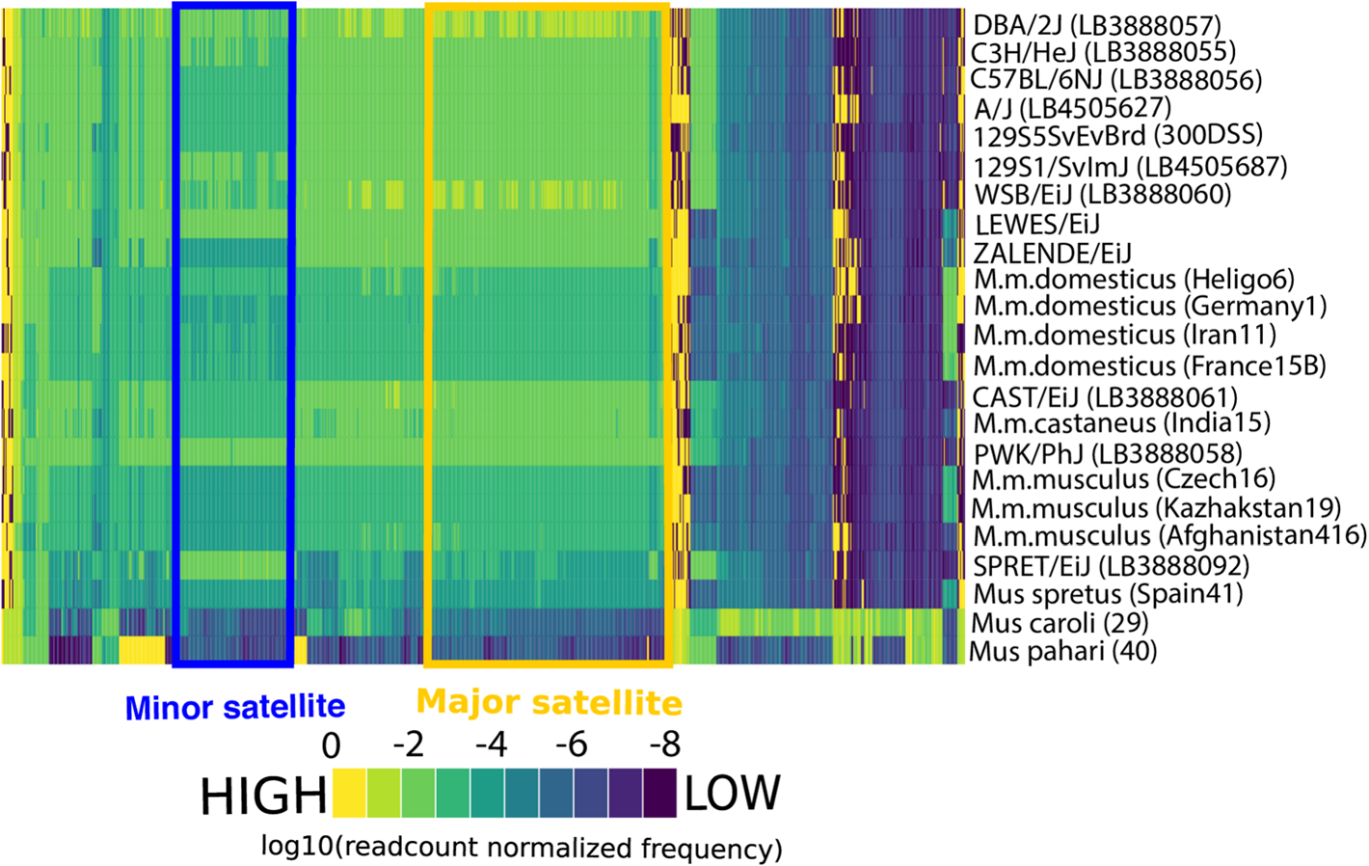
Consensus centromere 15-mers are the most abundant and the most variable 15-mers in diverse *Mus* genomes. Heatmap displaying the observed frequencies of the 1000 most variable 15-mers (columns) across a sample of diverse *Mus* genomes (rows). The color scale represents the normalized frequency of 15-mers. 15-mers present in the *Mus musculus* minor and major satellite consensus sequences are noted by the blue and yellow boxes, respectively

We first identified the most abundant 15-mers across a sample of 54 diverse mouse genomes. These genomes included common inbred mouse strains, wild-caught mice from multiple populations from each of the three principle house mouse subspecies (*M. m. domesticus*, *M. m. castaneus*, and *M. m. musculus*), and three divergent *Mus* taxa (*M. spretus, M. caroli, and M. pahari*). Consistent with prior reports [34], *M. musculus* minor and major centromere satellite 15-mers were among the most abundant *k*-mers in all surveyed *M. musculus* genomes (top 0.01% of all 15-mers). Centromere 15-mers were also among the most differentially abundant 15-mers across *M. musculus* genomes (Fig. 2, Supplementary Figure 3), a finding that hints at extensive centromere satellite copy number variation in this species.

*Mus spretus* shares an identical minor satellite consensus sequence with *Mus musculus* [27] and, as expected, exhibited a high abundance of minor satellite centromere 15-mers (Fig. 2, Supplementary Figure 3). In contrast, *Mus caroli* harbors divergent centromere satellite sequences from those in *M. musculus* [35]. We observed very weak enrichment for *M. musculus* major and minor consensus centromere 15-mer sequences in the *M. caroli* genome. Similarly, we found no enrichment for *M. musculus* major and minor centromere 15-mers in *M. pahari,* suggesting that *M. pahari* centromeres are also defined by a unique and divergent satellite.

### Strain and population-level variation in the abundance of *Mus musculus* consensus centromere satellites

Owing to the high prevalence and striking variability in the abundance of centromere satellite 15-mers among *Mus* genomes (Fig. 2, Supplementary Figure 3), we sought to further define strain, subspecies, and population variation in both major and minor satellite copy number. Below we present data for 31-mers (Fig. 1b), but again note that we observe qualitatively identical results for 15-mers (Supplementary Figure 1).

We first carried out pairwise comparisons between the distributions of minor and major satellite 31-mer frequencies in the surveyed inbred strain panel (Fig. 1b). Most pairwise comparisons are highly statistically significant for both satellite repeats (84/91 strain pairs with TukeyHSD, *P* < 0.05; Supplementary Table 1). Similarly, 22 of 28 wild-caught *M. musculus* population pairs exhibit significant differences in the frequencies of both minor and major satellite 31-mers (Supplementary Table 1; Tukey HSD, *P* < 0.01).

We next converted our normalized *k*-mer counts into absolute satellite copy number estimates to assess strain and population differences in consensus centromere size (see Methods). We estimate between 1320 and 260,220 minor satellite copies and 236,080–713,020 major satellite copies in the genomes of 14 inbred *M. musculus* strains. These copy number differences translate to minor (major) satellite array size differences of 3.96 kb – 780.66 kb (1.381 Mb – 4.171 Mb) per chromosome on average. Similarly, we estimate between 2900 and 37,240 minor satellite copies and 20,250–70,460 major satellite copies in wild-caught *M. musculus*, corresponding to minor and major satellite array size ranges of 8.7 kb – 111.7 kb and 118.4 kb – 412.2 kb per chromosome, respectively. The greater number of major satellite repeats relative to minor satellite repeats is consistent with the known size differences between the major and minor satellite array in *M. musculus* [26, 34, 36]. These estimates include only exact matches to the consensus satellite sequences and ignore the potential presence of other sequence elements that contribute to centromere size differences between samples. Nonetheless, the 197- (3-) fold range in absolute minor (major) consensus satellite copy number between closely related inbred *M. musculus* strains and the 13- (3-) fold range in absolute minor (major) consensus satellite copy number between wild caught *M. musculus* subspecies further suggests remarkable differences in centromere size between strains and subspecies.

We next sought to estimate the proportion of variation in both major and minor satellite copy number that is attributable to differences between inbred strains, rather than technical artifacts from library preparation and other sources of error. To this end, we modeled minor (major) satellite 31-mer counts as a function of strain identity and sequencing library and estimated the associated variance components. Over 80% of the variance in minor satellite 31-mer frequencies is due to strain differences (80.3%; *F*_13,168_ = 1940.25, *p* < 10^− 16^), whereas only 0.62% is attributed to variation between independent sequencing libraries (*F*_11,168_ = 17.94, *p* < 10^− 16^). Similarly, strain identity accounts for 56.4% of the variance in major satellite 31-mer frequencies (*F*_13,168_ = 1548.3, *p* < 10^− 16^), with library variation accounting for just 11% of the observed variance (*F*_11,168_ = 357.9, *p* < 10^− 16^). Thus, the majority of observed variation in both major and minor satellite 31-mer frequencies is due to intrinsic genomic differences between strains.

We adopted a similar ANOVA framework to estimate the proportion of variation in 31-mer frequencies that is explained by subspecies, population, and individual-level differences in wild-caught mice. Subspecies identity accounts for the majority of variation in minor satellite 31-mer frequencies (83.9% of variance; *F*_2,1836_ = 47,863.28, *p* < 10^− 16^), with only a minor contribution explained by variation between populations (1.3% of variance; *F*_5,1836_ = 300.36, *p* < 10^− 16^) or inter-individual differences within a population (2.6% of variance; *F*_50,1836_ = 58.32, *p* < 10^− 16^). The partitioning of variance in major satellite 31-mer frequencies follows a similar trend: 43.4% is explained by subspecies differences, 4.7% is attributable to population level differences, and 5.7% is due to inter-individual differences. We conclude that centromere satellite 31-mer frequencies are most differentiated between reproductively isolated subspecies, but only modestly differentiated between and within populations.

Although both major and minor satellite *k*-mer frequencies are influenced by strain and subspecies identity, there is only a weak correlation between the median major and minor satellite consensus copy numbers within genomes (adjusted *R*^*2*^ = 0.33, *P* = 0.05). Thus, samples with more major satellite copies do not necessarily have more minor satellite copies. Despite being physically linked, copy number variation at these two centromere satellite domains has evidently been shaped by distinct evolutionary and/or mutational processes.

Beyond strain, subspecies, and population-level variation, our dataset also reveals a striking difference in minor and major satellite *k*-mer frequencies between inbred strains and wild-caught mice. On average, inbred strains exhibit significantly higher satellite 31-mer frequencies than wild mice (Fig. 1b; Student’s *t*-test = 212.76; *P* < 2.2 × 10^− 16^). Indeed, Principle Component Analysis (PCA) of minor and major satellite 31-mer frequencies across diverse *M. musculus* samples identified inbred versus wild (i.e., outbred) as the major axes of differentiation (Supplementary Figure 4). This outcome is not an artifact of systematically undercounting centromere satellite *k*-mers with sequence mismatches to the consensus, as we also observe a reduced fraction of reads mapping to the centromere satellite consensus sequences in wild mice compared to inbred strains (Supplementary Figure 5). We speculate that inbreeding may lead to the expansion of centromere repeats in house mice, similar to observations and an earlier proposal for maize [37].

### Cytogenetic validation of strain differences in consensus centromere satellite abundance

**Fig. 3:**
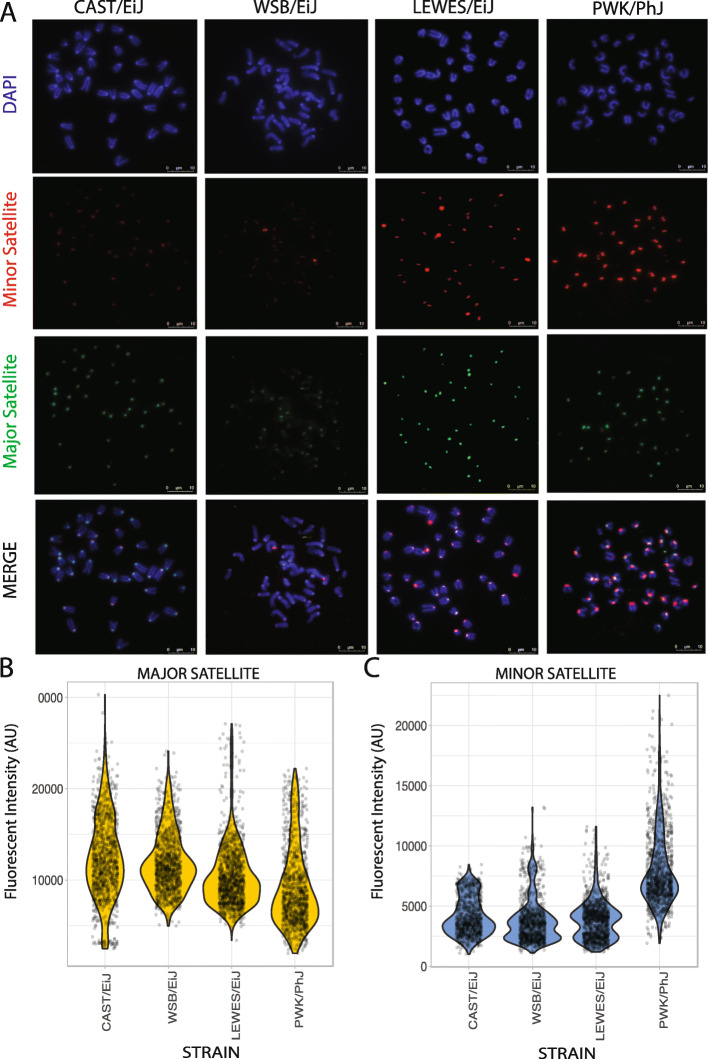
Quantitative FISH validates consensus centromere satellite copy number variation across inbred mouse strains. **a** Representative FISH images from four genetically diverse inbred strains: CAST/EiJ, WSB/EiJ, LEWES/EiJ, and PWK/PhJ. Individual color channels were manually manipulated using the Color Balance feature in FIJI for presentation purposes. Only raw, unedited images were used for quantification. Quantification of fluorescent intensity using DNA probes derived from the (**b**) major and (**c**) minor centromere satellite repeats across inbred strains. Points correspond to fluorescent intensity measurements for a single chromosome. A minimum of 40 centromeres from 36 cells were examined per strain. Fluorescent intensity is represented in arbitrary units (AU)

We used quantitative FISH (qFISH) to validate our *k*-mer based estimates of strain variation in consensus centromere satellite abundance (Fig. 3). We focused on a subset of strains that encompass a range of estimated minor satellite copy numbers and span three principal house mouse subspecies: CAST/EiJ (*M. m. castaneus*), WSB/EiJ (*M. m*. *domesticus*), LEWES/EiJ (*M. m. domesticus*), and PWK/PhJ (*M. m. musculus*) (Fig. 1b). We observed strong concordance between relative copy number and qFISH signals at both the minor and major centromere satellites (Fig. 3b). Notably, both *k*-mer and qFISH methods yielded a similar rank order of strains with respect to minor satellite abundance (median fluorescent intensity ranking in arbitrary units; WSB/EiJ = 3440 < CAST/EiJ = 3780 < LEWES/EiJ = 3820 < PWK/PhJ = 7055). Interestingly, in WSB/EiJ and LEWES/EiJ, several chromosomes consistently showed a higher minor satellite signal intensity relative to other chromosomes (Fig. 3a). This observation contrasts with the more uniform intensity of the minor satellite signal across all chromosomes in CAST/EiJ and PWK/PhJ (Fig. 3a). These findings point to chromosome-specific minor satellite accumulation and/or loss in some *M. m. domesticus* strains, highlighting an additional dimension of centromere diversity.

### Centromere satellite heterogeneity at the strain and population levels

Using *k*-mers with exact matches to the consensus sequences, we have uncovered significant variation in consensus centromere satellite copy number across diverse *M. musculus* samples. However, focusing on *k*-mers with exact matches to the consensus limits our ability to discover and analyze centromere repeat diversity within a genome. To study this important class of centromere variation, we estimated the average number of pairwise sequence differences between centromere satellite repeats in each *M. musculus* sample (see Methods). We refer to this metric as the *centromere diversity index* (CDI).

**Fig. 4:**
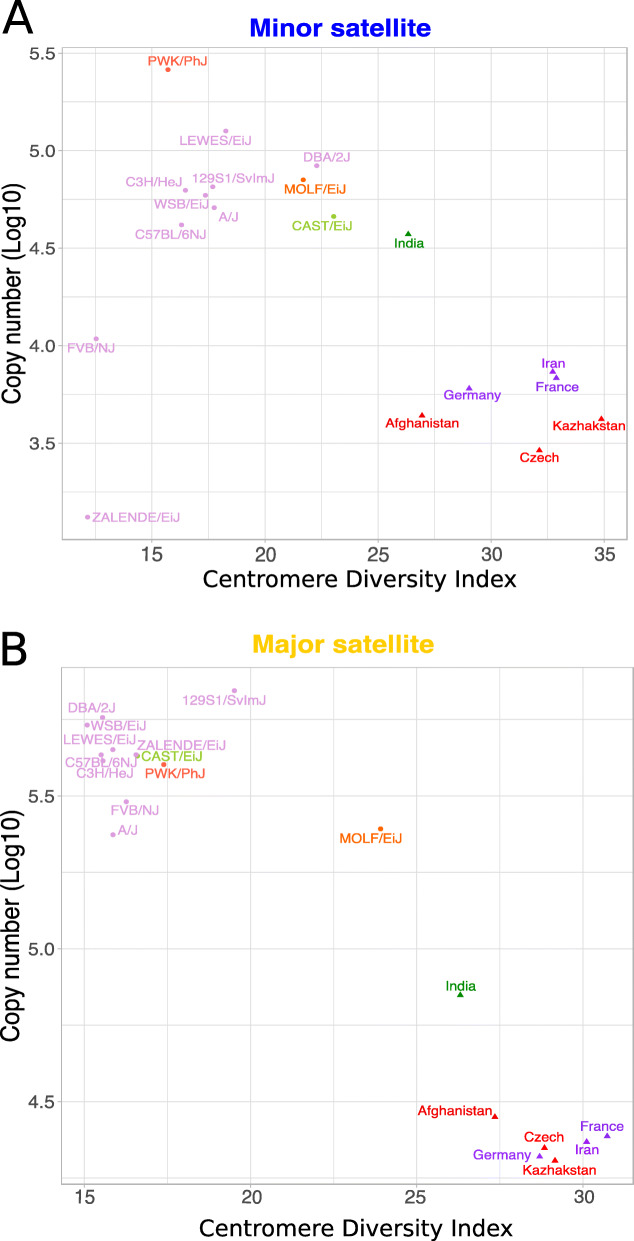
Negative correlation between centromere satellite copy number and sequence diversity in *Mus musculus*. Estimated centromere satellite copy number and centromere diversity index for the (**a**) minor or (**b**) major satellite sequence. Copy number was estimated from the median frequency of consensus centromere 31-mers in each sample. The three primary house mouse subspecies are denoted by different colors: red - *M. m. musculus*, purple - *M. m. domesticus*, and green - *M. m. castaneus*, orange – *M. m. molossinus*. Shapes distinguish inbred strains (circles) from wild-caught mice (triangles)

The minor satellite CDI is lower in inbred strains (range: 12.1–23.0) than in wild mice (range: 24.8–35.6). At the extremes, mice from the Kazhak population of *M. m. musculus* have minor satellite arrays that are nearly 3 times as diverse as those in the inbred strains ZALENDE/EiJ and FVB/NJ (Fig. 4a). For both inbred and wild mice, the average minor satellite CDI (inbred = 17.6 ± 3.49, wild = 30.2 ± 3.37) is slightly higher than the major satellite CDI (inbred = 16.9 ± 2.39, wild = 28.1 ± 2.02), despite the increased length and greater genomic abundance of the major satellite. We conclude that in *M. musculus* the minor satellite harbors appreciably higher sequence diversity than the major satellite (Fig. 4).

We next asked how variation in CDI is influenced by subspecies and population designation in wild mice. Pairwise comparisons of CDI values between subspecies and populations reveal significant differences at these levels of taxonomic organization (Tukey HSD for all pairwise tests; *P* < 0.05). Subspecies identity accounts for 39.2% of the variance in minor satellite CDI (F_2,15_ = 74.74, *P* < 10^− 15^), and population differences account for an additional 48.1% of the variance (F_4,15_ = 45.83, *P* < 10^− 16^). Variation in the major satellite CDI is most strongly influenced by subspecies origin (43.1%; F_2,15_ = 23.687, *P* < 10^− 8^), with a smaller contribution from population identity (13.4%; F_4,15_ = 3.70, *P* = 0.01).

### Relationship between centromere diversity and satellite copy number

Centromere diversity and consensus satellite copy number are highly variable across inbred strains and wild mice, prompting us to investigate the relationship between these two measures of centromere variation. Overall, there is a negative correlation between satellite copy number and CDI for both the minor and major satellite repeats (Fig. 4a and b; minor satellite: Spearman’s rho = − 0.40, *P* = 0.08; major satellite: Spearman’s rho = − 0.7, *P* = 0.001). Samples with high satellite copy number tend to have more homogenous repeats, whereas samples with lower satellite copy numbers tend to have greater repeat heterogeneity. This relationship is largely driven by the striking distinction between wild-caught mice and inbred strains. Relative to inbred strains, wild-caught mice harbor smaller and more diverse centromere arrays. The similarity in minor satellite size and diversity in inbred strain CAST/EiJ and wild-caught *M. m. castaneus* represents one possible exception to this pattern (Fig. 4a). We hypothesize that the inverse relationship between centromere satellite copy number and heterogeneity could be driven by differences in the opportunity for unequal crossing over. Homogenous repeats are more susceptible to unequal crossover events, leading to spontaneous repeat expansion and ultimately, large, homogenized repeat arrays [38]. In contrast, a repeat array with higher heterogeneity is expected to experience lower rates of unequal crossing over, leading to increased mutation accumulation at the locus and a self-perpetuated increase in repeat heterogeneity [38]. This hypothesis, combined with our observed differences between inbred strains and wild-caught mice, raise the possibility that phenomena specific to inbred strain genomes (or, potentially, the very process of inbreeding itself) may have influenced house mouse centromere architecture in pronounced ways.

### Sequence landscape of *Mus musculus* satellite diversity

Our CDI measure captures overall centromere satellite diversity within single genomes but does not pinpoint specific satellite sequence positions that are subject to high variability*.* To investigate the landscape of sequence polymorphisms along the major and minor centromere satellite repeats, we relaxed the criterion for perfect *k*-mer matching by considering all 15- and 31-mers with ≤ 2 and ≤ 5 mismatches, respectively, from the *M. musculus* centromere satellite consensus sequences. These relaxed edit-distance *k*-mers map to regions of the mm10 reference genome with extremely limited frequency, yielding minimal background noise from non-centromere regions (Supplementary Table 2). In addition, this larger set of *k*-mers can be unambiguously assigned to positions in the minor and major satellite consensus sequences allowing us to quantify the proportion of *k*-mers harboring nucleotide mismatches at each site. Using the percentage of non-consensus nucleotides at each position, we then identified sites with variable nucleotide usage across samples.

**Fig. 5:**
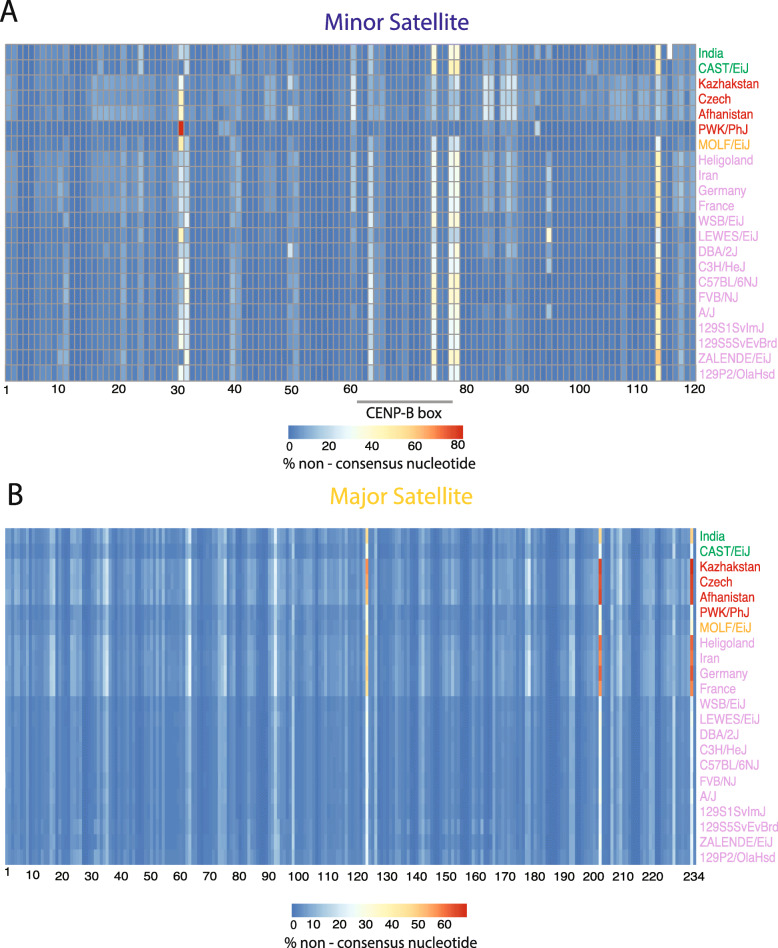
Landscape of nucleotide variation across centromere satellite repeats. Heatmap of non-consensus nucleotide usage for positions in the (**a**) minor satellite consensus sequence and (**b**) major satellite consensus sequence. Each row corresponds to a single sample with sample names colored by subspecies origin: green – *M. m. castaneus*, red – *M. m. musculus,* purple – *M. m. domesticus,* orange – *M. m. molossinus*

Overall, sequence diversity is not uniformly distributed across the minor and major satellite sequences, but instead restricted to a limited number of sites that are variable between genomes (Fig. 5). Despite its smaller size, the minor satellite harbors more sites with at least 20% non-consensus nucleotide usage than the major satellite (107 versus 79; Fig. 5a and b). Although divergence from the satellite consensus is concentrated at a minority of sites, different samples vary in the frequency of non-consensus nucleotides present at a given position. For example, LEWES/EiJ, WSB/EiJ and 129S1/SvImJ have similar CDIs (CDI = 17–18), but their minor satellite sequence landscapes are distinct (Fig. 5a).

Intriguingly, three positions around the CENP-B (Centromere protein B) binding motif of the minor satellite (positions 62–78) show high levels of nucleotide variability among *M. musculus* [39]. In particular, we identified high nucleotide variability at two minor satellite positions within the CENP-B binding region (positions 75 and 78) and at one position immediately adjacent to the binding motif (position 79) (Supplementary Figure 6). Remarkably, despite incredible variation in nucleotide usage across inbred strains and wild-caught mice (Supplementary Figure 6), position 75 is critical for CENP-B binding in human centromere satellite DNA [40]. Overall, we estimate that approximately 45% of mouse minor satellites do not contain the functionally critical “G” nucleotide at position 75, and therefore do not facilitate CENP-B binding. In addition, position 78 exhibits more variability in the proportion of non-consensus nucleotide usage than any other minor satellite position in both inbred strains and wild-caught mice. Prior investigation has established that CENP-B binding is important, albeit dispensable, for kinetochore assembly and chromosome segregation [8]. However, more recent work has posited a role for CENP-B in both CENP-A assembly and heterochromatin formation, and in counteracting functional differences between heterozygous centromeres, thereby attenuating the potential for meiotic drive [41, 42]. We speculate that the observed variation in and around the CENP-B box could influence CENP-B binding efficiency across diverse *M. musculus* strains and wild populations, a possibility that merits future functional investigation in the context of meiotic drive.

We also uncover clear differences in the satellite sequence landscape between wild-caught mice and inbred strains. On average, inbred strains have lower rates of non-consensus nucleotide usage (minor satellite 2.9–4.9%; major satellite 3.6–4.4%) compared to wild-caught mice (minor satellite 4.8–6.8%; major satellite 5.7–6.5%). This finding aligns with the higher CDI observed in wild-caught compared to inbred mice, lending further support to the conclusion that wild-caught *M. musculus* have more diverse and heterogenous centromere satellites than inbred strains.

### Phylogenetic distribution of minor satellite copy number and repeat heterogeneity

**Fig. 6:**
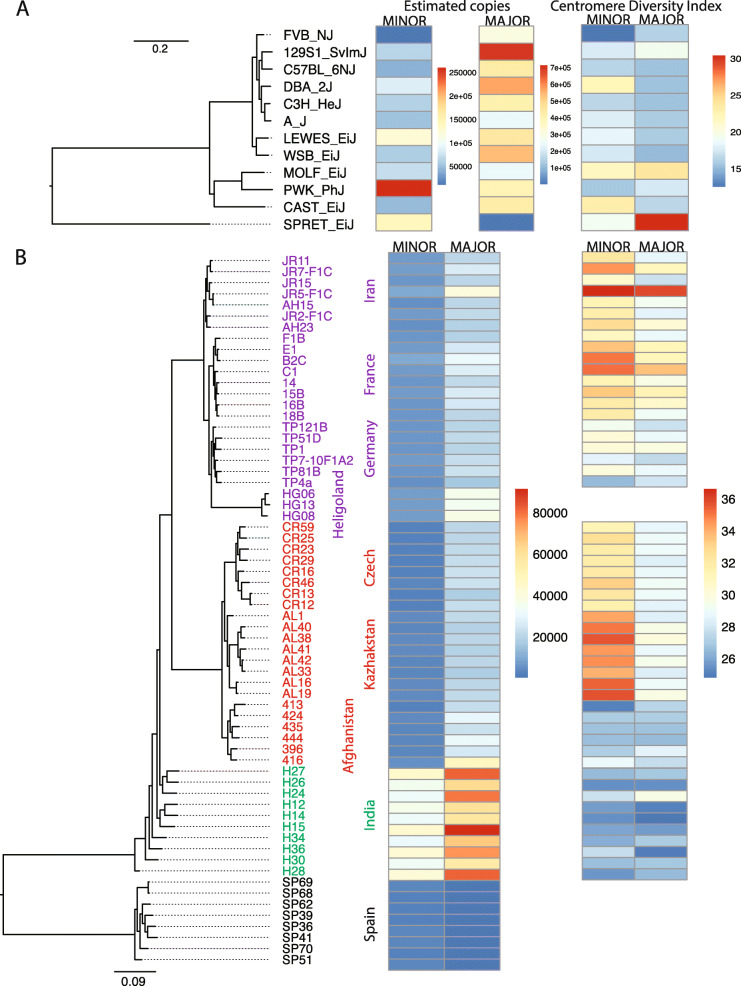
Phylogenetic distribution of centromere satellite copy number and satellite heterogeneity in inbred strains and wild mice. **a** Maximum likelihood phylogenetic tree for 11 inbred house mouse strains and the outgroup, SPRET/EiJ. For each strain and for both the major and minor satellites, estimated satellite copy number and CDI are indicated by boxes shaded according to the corresponding color scales to the right of each heatmap. **b** Maximum likelihood phylogenetic tree for wild *M. musculus* samples and the outgroup of *Mus spretus* samples. The mouse subspecies and species are denoted by different colors: red - *M. m. musculus*, purple - *M. m. domesticus*, green - *M. m. castaneus* and black - *M. spretus*

To investigate how centromere architecture evolves in house mice, we analyzed the distribution of centromere diversity metrics in a phylogenetic framework [43]. We quantified the proportion of variation in major and minor satellite copy number and CDI that is explained by the phylogenies relating inbred strains (Fig. 6a) and wild-caught mice (Fig. 6b). If variation in a given satellite diversity metric is well-predicted from the evolutionary relationships among samples, the metric should exhibit a high phylogenetic heritability, ${H}_P^2$. In contrast, if centromere copy number or CDI evolve at exceptionally high rates, these measures of centromere variation may become decoupled from the signal of shared descent among organisms and exhibit a weak phylogenetic heritability (i.e., low ${H}_P^2$). Owing to the stark differences in their centromere architecture and breeding history, inbred strains and wild-caught mice were analyzed independently.

Across inbred strains, the phylogenetic heritability of both minor satellite copy number (${H}_P^2$ = 0.56; *P* = 0.15) and CDI (${H}_P^2$ = 0.15; *P* = 0.21) is low, and not significantly different from zero. Evidently, both measures of minor satellite variation evolve sufficiently rapidly to outpace signals of strain relatedness. In contrast, variation in both major satellite copy number (${H}_P^2$ = 0.98; *P* = 0.24) and CDI (${H}_P^2$ = 0.99; *P* = 0.07) exhibited a high, albeit non-significant, phylogenetic heritability. Although modest sample sizes limit the power of this analysis, the absolute differences in the ${H}_P^2$ estimates between the minor and major satellites align with the conclusion that these two centromere satellites evolve via distinct regimes, potentially mediated by differences in selective pressures or mutational mechanisms.

In wild mice, variation in both satellite copy number and satellite heterogeneity were well-predicted by the evolutionary relationships among samples (minor satellite copy number ${H}_P^2$ = 0.99; *P* = 0.003, major satellite copy number ${H}_P^2$ = 0.98; *P =* 0.003, minor satellite CDI ${H}_P^2$ = 0.98; *P* = 0.0009, major satellite CDI ${H}_P^2$ = 0.89; *P =* 0.01). The contrast in minor satellite ${H}_P^2$ estimates between the inbred and wild-caught mice provides further support for the hypothesis that inbreeding fosters a unique setting for the evolution of centromere architecture.

### Assessing the phenotypic consequences of centromere diversity in *Mus musculus*

Centromere integrity is essential for genome stability and if not maintained can lead to cancer and infertility [6–10]. We next asked whether observed centromere satellite diversity influences the stability of genome transmission. Using publicly available phenotype data from the Mouse Phenome Database (https://phenome.jax.org/) we searched for correlations between centromere satellite copy number and micronuclei formation, a hallmark of chromosome instability [44, 45]. We found no significant correlation between this measure of genome stability and either major or minor satellite consensus copy number (Supplementary Figure 7). However, small sample sizes, uncertainty in our copy number estimates, and imprecision in the chromosomal instability phenotype may conceal true functional links.

Centromeres are reservoirs for the accumulation of selfish drive elements that can hijack the inherent asymmetry of female meiosis to bias their own transmission into the oocyte [24, 46, 47]. We next asked whether centromere satellite copy number differences among inbred strains lead to systematic meiotic drive in diverse mouse populations. We profiled datasets from the Diversity Outbred (DO) mouse population, a heterogenous stock population founded from 8 strains with distinct centromere satellite copy number states [48]. We scanned genotypes of DO mice from 12 successive generations of outbreeding for evidence of over-transmission of centromere-proximal alleles from one (or more) founder strain(s). We found no evidence for non-Mendelian transmission of centromere-proximal regions in the DO (Supplementary Figure 8). This result suggests (i) the absence of centromere-mediated meiotic drive in this complex population, (ii) the lack of power to detect weak drive signals, (iii) that drive is influenced by multiple genetic factors [49], or (iv) that aspects of centromere architecture other than minor satellite copy number may be critical for defining drive potential.

## Discussion

Evolutionary theory predicts that genomic regions with key cellular roles should exhibit reduced rates of evolution in order to preserve their biological function. Centromeres are paramount for chromosome segregation and the maintenance of genome stability, but, paradoxically, centromere satellite sequences are known to evolve rapidly between species [17, 50–52]. Despite this knowledge, comparatively little is known about the extent of centromere variation over shorter evolutionary timescales, including at the population level. Here, we developed a powerful *k*-mer based workflow for quantifying centromere satellite copy number and sequence diversity from whole genome sequence data. We apply this analytical framework to 100 genomes from diverse inbred and wild-caught mice to characterize multiple dimensions of mouse centromere variation.

We discovered key differences in the mode and rate of evolution of the *M. musculus* major and minor satellite sequences. Most notably, minor satellite arrays exhibited more extreme variation in copy number and CDI in comparison to the major satellite arrays (Fig. 1b and Fig. 4). These findings presumably manifest from the distinct biological functions of the major and minor satellite domains. The major satellite repeat forms the pericentromeric heterochromatin and is responsible for the establishment and maintenance of sister chromatid cohesion [1]. The minor satellite repeat binds to CENP-A, a specialized centromeric histone variant responsible for kinetochore complex specification and assembly [1]. In many animal species, CENP-A is rapidly evolving, which imposes a complementary selection pressure on the centromere satellite sequence to ensure protein-DNA compatibility [53–55]. The CENP-A amino acid sequence is perfectly conserved among *M. musculus* subspecies, but sequence diversity at the centromere satellite could influence the efficiency of CENP-A binding, with potential downstream consequences for kinetochore assembly and chromosome segregation [22, 56]. The co-evolutionary dynamics between the minor satellite DNA and important kinetochore proteins have likely contributed to the accelerated evolution of the minor satellite relative to the major satellite, which does not serve as a sequence substrate for kinetochore proteins. Indeed, at least in inbred strains, we show that shared evolutionary history is a poor predictor of minor satellite copy number and sequence heterogeneity, suggesting that minor satellite arrays evolve sufficiently rapidly to outstrip signals of recent shared descent. In contrast, strain relatedness is a stronger predictor of both copy number and sequence heterogeneity across the major satellite, although our analysis lacks sufficient power to obtain statistically significant results (Fig. 6c).

Our genomic survey also reveals pronounced differences in centromere satellite copy number between inbred mouse strains. ZALENDE/EiJ, a wild-derived inbred strain of *M. m. domesticus* that harbors numerous Robertsonian chromosomal fusions, carries the lowest minor satellite *k*-mer frequencies of any sample in our survey. This finding reinforces conclusions from prior investigations of centromere size in this strain [22, 24]. Interestingly, ZALENDE/EiJ does not exhibit a parallel decrease in the amount of major satellite DNA relative to other inbred strains, indicating that the mechanism of Robertsonian fusion only leads to the loss of significant amounts of minor satellite DNA. At the other extreme, PWK/PhJ, a wild-derived inbred strain of *M. m. musculus*, has the greatest abundance of minor satellite *k*-mers. This strain is divergent from the *M. m. domesticus*-derived centromere consensus sequence, suggesting that our exact-match *k*-mer estimate may undercount centromere-derived *k*-mers from this genome, leading to a downwardly biased estimate of centromere size in this strain. Indeed, the proportion of sequenced reads from this strain that map to the consensus minor satellite sequence is greater than expected based on the frequency of exact match *k*-mers (Supplementary Figure 5). Beyond these extreme outliers, median minor satellite *k*-mer frequencies still span a 10-fold range between closely related inbred mouse strains, implying a potential 10-fold size difference in the core CENP-A binding centromere region. CENP-A ChIP-seq and cytogenetic experiments in diverse strains are needed to determine whether CENP-A recognizes potential centromere-embedded sequences other than the minor satellite, and to assess whether strains vary in the proportion of the minor satellite array that is CENP-A binding.

We also uncover significant subspecies variation in centromere copy number among wild-caught *M. musculus*. As in the inbred strains, there is greater variation in minor as opposed to major satellite abundance among wild house mice. *M. m. castaneus* harbors the highest frequency of minor satellite *k*-mers, followed by *M. m. domesticus* and *M. m. musculus*. We observe only modest variation for both major and minor satellite *k*-mer frequencies between and within populations of wild-caught animals. These findings suggest that differences in centromere size are potentially reinforced through reproductive isolation between subspecies, whereas levels of gene-flow between house mouse populations are sufficient to counteract the emergence of large population differences in centromere size.

In addition to centromere size, we also uncover significant variation in the magnitude of satellite heterogeneity among inbred strains and among wild-caught house mouse populations. On average, across inbred strains, any two minor satellite repeats differ at ~ 17 sites, corresponding to ~ 14% nucleotide divergence. These values are notably higher for wild-caught mice (minor satellite: ~ 30 variable sites, ~ 25% nucleotide divergence). Our *k*-mer based and consensus mapping strategies are agnostic to chromosome of origin, begging the question of whether satellite repeats are more similar within versus between house mouse chromosomes. At the very least, it is clear that there are striking chromosome-level differences in minor satellite array size among some inbred strains (Fig. 3). On-going long-read sequencing projects for house mice may yield the needed data to address this question. Nonetheless, while it has been widely assumed that inbred house mouse centromeres are highly homogenous, our findings call into question whether this is a fair statement.

Our work also identified surprising differences in centromere satellite architecture between wild-caught and inbred mice. Wild-caught mice exhibit lower major and minor centromere satellite copy numbers and greater satellite heterogeneity than the inbred strains (Fig. 5). Similar observations have been previously reported for centromeres in inbred and outbred maize [37]. Together, these findings suggest that the inbreeding process itself might drive the homogenization of satellite arrays and facilitate the fixation of larger centromeres. Indeed, prior studies have established that larger centromeres may recruit more kinetochore proteins than smaller centromeres, enabling larger centromeres to selfishly bias their own segregation into the oocyte during asymmetric female meiosis, a process known as centromere drive [22, 24, 57]. In the context of inbreeding, such “strong centromeres” should be rapidly fixed. Recurrent bouts of de novo centromere expansion and fixation could lead to rapid, run-away amplification of centromere satellites in bottlenecked inbreeding populations compared to large, randomly mating wild mouse populations. Thus, centromere size and repeat heterogeneity within inbred strains may not faithfully capture the native state of *M. musculus* centromeres. Future investigations that chronical changes in centromere size from the earliest stages of inbreeding onward could provide a real-time window into the mutational processes that promote this architectural shift.

Our analyses define the extent of centromere copy number and sequence diversity in diverse inbred strains, motivating investigation into the phenotypic consequences of this variation. As an initial attempt to address this outstanding challenge, we looked for correlations between satellite copy number and a phenotype proxy for chromosome instability: the frequency of spontaneous micronuclei formation in peripheral blood cells. We observed no significant relationship between these variables, although the tested phenotype – spontaneous micronuclei formation – is likely an imprecise measure of centromere-mediated genome instability [45]. Furthermore, our analysis was limited to a small number of inbred strains with available published data and is underpowered to find small to moderate strength genotype-phenotype correlations. We also tested whether variation in minor satellite copy number leads to centromere drive in a mouse population developed from eight inbred strains with variable minor satellite copy numbers [48]. We found no evidence for strong non-Mendelian transmission of centromere-proximal variants although again, our analysis likely suffers from a lack of statistical power to find weak to moderate drive signals. By providing the first quantitative estimates of centromere satellite diversity in a panel of widely used inbred strains, our investigation critically informs strain choice for future studies that aim to rigorously and explicitly test how centromere diversity influences the fidelity of chromosome segregation and genome stability.

Ultra-long read sequencing technologies are now enabling sequence-level resolution of mammalian centromeres [19–21, 58]. However, the high cost of these methods and their labor-intensive analyses put their use out of reach for most investigators and effectively limit the number of population samples that can be analyzed. Our powerful *k*-mer-based workflow for assaying the architectural and sequence diversity of centromeres circumvents these critical limitations and is readily extendable to large numbers of genomes. Using only short read data in public repositories, our work has provided key evolutionary insights into the scope of population and subspecies variation across house mouse centromeres, establishing the needed foundation for functional tests of centromere diversity in an important biomedical model system.

## Conclusions

Our study presents the largest survey of population-level centromere variation in mammals to date, encompassing both inbred and wild mice from three cardinal *M. musculus* subspecies. We provide the first quantitative estimates of centromere size and satellite heterogeneity between house mouse subspecies, populations, and inbred strains, uncovering significant variation at each of these taxonomic levels. We show that the major and minor satellite arrays in *M. musculus* exhibit distinct patterns of diversity, consistent with the action of unique evolutionary pressures at these two functionally distinct satellite domains. We further identify striking differences in centromere architecture between inbred strains and wild-caught mice, suggesting that inbreeding actively leads to the expansion and homogenization of centromere satellites. Taken together, our work presents a powerful bioinformatic framework for probing centromere diversity that can be readily extended to other taxa and adapted to interrogate diversity at other satellite rich genomic regions. Moreover, our analyses yield a catalog of centromere diversity across diverse mice that will guide future investigations on the functional consequences of centromere variation in mammals.

## Materials and methods

### Whole genome sequencing data

Illumina whole genome sequences from 100 house mouse (*Mus*) genomes were obtained in bam and fastq formats from public repositories (Supplementary Table 3). These samples include 33 inbred house mouse strains of predominantly *Mus musculus domesticus* ancestry [32], 27 wild *M. m. domesticus* mice from four populations, 22 wild *M. m. musculus* from three populations, ten wild *M. m. castaneus* from India, eight wild *Mus spretus* from Spain [33], a wild-derived inbred strain of *Mus caroli* (CAROLI/EiJ), and a wild-derived inbred strain of *Mus pahari* (PAHARI/EiJ) [31]. *M. caroli* and *M. pahari* sequence reads were mapped to the *M. musculus* reference (mm10) using bwa mem version 0.7.9 [59]. Optical duplicates were removed using the *rmdups* command in samtools version 1.8 [60].

### *k*-mer frequencies and normalization

We computed the observed frequency of all *k*-mers in each mouse genome on a per-library basis. Briefly, each sequenced read in a sample’s fastq file was decomposed into its constituent nucleotide words of length *k*, or *k*-mers, using a custom Python script (KmerComposition.py). We selected two lengths for *k*: *k* = {15, 31}. These *k* values were selected to balance computational speed (*k* = 15) and provide high sequence specificity (*k* = 31). Each analyzed genome captured 440–965 million unique 15-mers and 1.1–14.5 billion unique 31-mers.

The efficiency of PCR amplification is not uniform with respect to GC-content, and this can lead to biases in the nucleotide composition of sequencing libraries [61]. If uncorrected, such biases could cause false inference of differences in *k*-mer abundance between independent libraries and samples. We implemented a GC-correction to rescale raw *k*-mer counts by the extent of the observed GC-bias in each library. Briefly, we randomly selected a set of ~ 100,000 *k*-mers that occur uniquely in the mouse reference genome (mm10). For each sample, we modeled the observed counts of these unique *k*-mers as a function of their GC-content using LOESS regression, with the span parameter set to 0.4. The LOESS regression produced a predicted *k*-mer count for each GC-content bin; these values correspond to the magnitude and direction of the empirical GC-bias in the sequencing library and represent the expected “amplification” of a *k*-mer based on its GC-content. Finally, observed *k*-mer frequencies were normalized by the LOESS predicted count for the corresponding GC-content bin:

$$ \mathrm{Normalized}\ k-\mathrm{mer}\ \mathrm{count}=\log 10\frac{\mathrm{observed}\ k-\mathrm{mer}\ \mathrm{count}}{\mathrm{LOESS}\ \mathrm{predicted}\ k-\mathrm{mer}\ \mathrm{count}}, $$

Normalized values were used for comparisons across libraries and samples.

We used reads derived from multiple independent sequencing libraries from a single inbred strain to confirm that our strategy was robust to potential artifacts introduced during library preparation. After GC-correction, we observe excellent concordance of centromere *k*-mer frequencies among replicate libraries for a given strain (Pearson correlation 0.990 < R^2^ < 0.999; Supplementary Figure 2). As expected, concordance between strains was generally weaker (Pearson correlation 0.41 < R^2^ < 0.998; Supplementary Figure 2).

### Identification of highly variable *k*-mers across house mouse

To identify *k*-mers that differ in abundance across genomes, we selected a representative subset of *n* = 54 diverse *Mus* samples (see Supplementary Table 3) and computed the variance in observed 15-mer frequencies across their genomes:

$$ \frac{\sum \limits_{s=1}^n{\left({F}_s-\overline{F}\right)}^2}{n-1}, $$

where *F* is the absolute 15-mer frequency standardized by the read depth of strain *s* and $\overline{F}$ is the average normalized frequency of the 15-mer across the selected 54 strains. The 1000 15-mers with the largest variance were plotted as a heatmap using the R package *pheatmap*.

### Quantifying centromere satellite abundance

We used a reference-informed approach to quantify the relative copy number of centromere satellites in each mouse genome. We first decomposed the minor and major satellite consensus sequences into their constituent *k*-mers [26]. We then queried the GC-corrected frequency of these centromere *k*-mers in each analyzed library and compared the distribution of these centromere *k*-mer frequencies across libraries and samples.

The relative copy number of a centromere satellite consensus sequence in a given mouse genome was estimated from the median frequency of all constituent *k*-mers. For example, if the median log_10_ GC-corrected count for *k*-mers present in the major satellite in a given genome was 5, we estimated 10^5^ copies of the major satellite in that genome. This quantity is highly correlated with the overall percentage of sequenced reads that map to the minor and major consensus sequences (Pearson correlation; minor: R^2^ = 0.73, *P* = 2.64 × 10^− 5^; major: R^2^ = 0.85, *P* = 1.65 × 10^− 7^; Supplementary Figure 5), suggesting that it provides a faithful readout of centromere satellite copy number.

We observe little variation in centromere satellite copy number among wild-caught mice sampled from a single population and replicate sequencing libraries from a single inbred strain (Supplementary Figure 9). A subset of our analyses therefore combined all individuals from a given population to produce a single population-level copy number estimate. Similarly, for inbred strains with multiple sequencing libraries, GC-corrected *k*-mer counts were aggregated across libraries.

### Quantifying within genome centromere satellite diversity

To quantify centromere satellite diversity within a genome, we computed the average number of sequence differences between independent satellite repeats, a metric we term the *centromere diversity index* (CDI). We first mapped sequenced reads to the major and minor centromere consensus sequences using bwa version 0.7.9 [59]. We then partitioned reads using samtools version 1.8 [60] based on (i) whether they mapped to the major or minor satellite, (ii) whether they mapped to the forward or reverse strand to prevent comparing sequences to their reverse complement, and (iii) their mapped position along the consensus sequence. For each pair of reads mapping to an identical site in the same orientation on the major or minor satellite sequence, we computed the average number of observed sequence differences, *d*_*ij*_. We then derived the CDI by averaging over all *N* tested read pairs:

$$ \mathrm{Centromere}\ \mathrm{Diversity}\ \mathrm{Index}\ \left(\mathrm{CDI}\right)=\frac{\sum \limits_{strand}\sum \limits_L\sum \limits_{ij}{d}_{ij}}{N} $$

where *L* is the length of the satellite repeat unit (*L* = 120 and *L* = 234 for the minor and major satellites respectively).

### Variance component estimation

We adopted an ANOVA framework to estimate the proportion of variation in satellite copy number and CDI that was explained by various levels of taxonomic organization. Major and minor satellite values were analyzed independently. For inbred strains, we modeled *k*-mer counts and CDI as a function of strain identity and library. For wild mice, we modelled *k*-mer counts and CDI as a function of subspecies, population, and sample. For each ANOVA, a Tukey HSD post hoc test was applied to evaluate the significance of pairwise comparisons between groups. All analyses were performed in R using the baseR aov and TukeyHSD functions.

### Quantifying consensus centromere satellite polymorphisms

To summarize the sequence polymorphism landscape across centromere satellite repeats, we identified *k*-mers with a fixed edit distance (*h*) of the minor and major satellite consensus sequence. For *k* = 15, we allowed *h* ≤ 2, and for *k* = 31 we allowed *h* ≤ 5. We used the frequencies of these relaxed edit distance *k*-mers, in conjunction with their positions across their respective satellite consensus sequences, to derive a vector of relative nucleotide probabilities for each position in the satellite consensus sequence. At a given position, we computed the total frequency of *k*-mers with an “A”, “C”, “G”, or “T” at the focal position. These per-nucleotide *k*-mer frequencies were then converted to relative probabilities summing to one and used to populate a 4x*N* “polymorphism matrix” for each analyzed sample, where *N* = 120 for the minor satellite sequence and *N* = 234 for the major satellite. Note that this approach ignores the contribution of indel mutations to sequence polymorphism at centromere satellite repeats. We then compared the percentage of non-consensus nucleotides for each strain across the minor and major consensus satellite sequence.

### Phylogenetic analysis of centromere diversity

Maximum likelihood trees for inbred strains and wild-caught mice were independently constructed using RAxML version 8.2.12 [62]. The phylogenetic tree for inbred strains was constructed from 56,500,187 high quality SNPs identified in 12 inbred *M. musculus* genomes and the outgroup SPRET/EiJ. The phylogenetic tree for wild mice was constructed from 1,547,278 high quality SNPs from 78 *M. musculus* and *M. spretus* genomes. To create each tree, an initial set of 20 ML trees was constructed using the GTRGAMMA substitution model. These trees were used as input for subsequent branch length and topology refinements in order to estimate the tree with the highest likelihood. We then used *GTRCAT* to derive bootstrap support values for the best ML tree, with the number of random seeds set to 12,345.

We applied Lynch’s phylogenetic comparative method to estimate the phylogenetic heritability of centromere satellite copy number and CDI [43]. Under a neutral (i.e., Brownian motion) model of evolution, the extent of phenotypic divergence between samples should be proportional to their genetic divergence. We computed phylogenetic variance-covariance matrices from the inbred strain and wild caught *M. musculus* ML phylogenies and then used these matrices to estimate the proportions of variation in both major and minor satellite copy number and CDI that are explained by the underlying trees. These quantities were then divided by the total variance in satellite copy number and CDI to calculate the phylogenetic heritability (${H}_P^2$) of each diversity parameter. The significance of observed values was assessed by an ad hoc permutation test. We shuffled observed satellite copy number and CDI values across the tree tips and then re-estimated ${H}_P^2$ on each permuted dataset. Empirical *P*-values were determined from the quantile position of the observed ${H}_P^2$ value along the distribution of 1000 permuted values.

All analyses were performed in R using the Analysis of Phylogenetics and Evolution (ape v5.3) package [63].

### Animal husbandry

The following inbred mouse strains were obtained from The Jackson Laboratory: CAST/EiJ, LEWES/EiJ, PWK/PhJ, WSB/EiJ. Mice were housed in a low barrier room and provided food and water ad libitum. Mice were euthanized by CO_2_ asphyxiation or cervical dislocation in accordance with recommendations from the American Veterinary Medical Association.

### Mouse embryonic fibroblasts cultures

Three to seven primary mouse embryonic fibroblast (MEF) lines were isolated from E12.5-E13.5 embryos from four inbred strains: CAST/EiJ, LEWES/EiJ, PWK/PhJ, and WSB/EiJ. We used a total of four pregnant female mice (one from each inbred strain) to obtain enough MEF lines for the biological replicates required for the experiment. MEFs were cultured in MEF media composed of Dulbecco’s Modified Eagle medium (DMEM) supplemented with 10% FBS (Lonza), 100μg/mL Primocin (Invivogen) and 1xGlutaMAX (Thermo Fisher Scientific/GIBCO). MEFs were cultured in 150 mm tissue culture-treated plates (Thermo Fisher Scientific) at 37 °C in a humidified atmosphere with 5% CO_2_.

### Metaphase chromosome spreads and FISH

MEFs were used for the preparation of metaphase spreads. Briefly, MEFs were cultured in MEF media to ~ 80% confluency at 37 °C in a humidified atmosphere with 5% CO_2_ in MEF media. Cells were subsequently serum starved on MEF media without FBS and exposed to 0.02 μg/ml Colcemid (Thermo Fisher Scientific/GIBCO) for 12 h to synchronize and arrest cells in metaphase. MEFs were subsequently shaken off and resuspended in hypotonic solution (56 mM KCl) for 60 min. The harvested cells were then gradually fixed in 3:1 Methanol:Glacial Acetic Acid under constant agitation. Cells were pelleted by centrifugation, the fixative decanted off, and re-fixed for a total of 3–4 times. Following the final fixation round, cells were suspended in a 1–2 mL volume of fixative and dropped onto slides from a height of ~ 1 m. Slides were allowed to air dry for approximately 10 min and then stored at -20C until hybridization.

Commercially synthesized oligos corresponding to the *M. musculus* major and minor satellite sequences were PCR amplified and fluorescently labelled via nick translation. Primer sequences are listed in Supplementary Table 4. Briefly, 250–1000 ng of PCR-amplified DNA was combined with nick translation buffer (200 mM Tris pH 7.5, 500 mM MgCl_2_, 5 mM Dithiothreitol, 500 mg/mL Bovine Serum Albumin), 0.2 mM dNTPs, 0.2 mM fluorescent nucleotides, 1 U DNAse (Promega) and 1 U DNA Pol I (Thermo Fisher Scientific). One of three fluorescent nucleotides was used for each satellite probe set: Fluorescein-12-dUTP (Thermo Fisher Scientific), ChromaTide Texas Red-12-dUTP (Thermo Fisher Scientific/Invitrogen), and Alexa Fluor 647-aha-dUTP (Thermo Fisher Scientific/Invitrogen). The reaction mixture was incubated at 14.5 C for 90 min, and then terminated by addition of 10 mM EDTA. Probes ranged from 50 to 200 bp in size, as assessed by gel electrophoresis.

Probes were used in FISH reactions on MEF metaphase cell spreads. Probes were denatured in hybridization buffer (50% formamide, 10% Dextran Sulfate, 2x saline-sodium citrate (SSC), mouse Cot-1 DNA) at 72 °C for 10 min and then allowed to re-anneal at 37 °C until slides were ready for hybridization. Slides were dehydrated in a sequential ethanol series (70, 90, 100%; each 5 min) and dried at 42 °C. Slides were then denatured in 70% formamide/2x SSC at 72 °C for 3 min, and immediately quenched in ice cold 70% ethanol for 5 min. Slides were subjected to a second ethanol dehydration series (90, 100%; each 5 min) and air dried. The probe hybridization solution was then applied to the denatured slide. The hybridized region was then cover-slipped and sealed with rubber cement. Hybridization reactions were allowed to proceed overnight in a humidified chamber at 37 °C. After gently removing the rubber cement and soaking off coverslips, slides were washed 2 times in 50% formamide/2x SSC followed by an additional 2 washes in 2x SSC for 5 min at room temperature. Slides were counterstained in 0.05 ng/mL DAPI (Thermo Fisher Scientific/Invitrogen) for 10 min and air dried at room temperature. Lastly, slides were mounted with ProLong Gold AntiFade (Thermo Fisher Scientific/Invitrogen) and stored at -20C until imaging.

### Image capture and fluorescence intensity quantification

FISH reactions were imaged at 63x magnification on a Leica DM6B upright fluorescent microscope equipped with fluorescent filters (Leica model numbers: 11504203, 11504207, 11504164), LED illumination, and a cooled monochrome Leica DFC7000 GT 2.8 megapixel digital camera. Images were captured using LAS X (Version 3.7) at a resolution of 1920 × 1440 pixels. Images were collected at a plane with maximal intensity using consistent exposure settings across slides (DAPI at 20 ms, TxRed at 50 ms and FITC at 200 ms). The mean intensities of FISH signals at each centromere were calculated in areas drawn around centromeres based on thresholding with background subtraction. Signals were quantified from all centromeres within a cell (*n* = 40). FISH fluorescent intensity signals were collected from two independent cell lines (biological replicates) from each strain and two independent experiments were conducted for each cell line with fluorophores swapped for each sequence (technical replicates). We collected images from 8 to 10 cells per replicate, amounting to > 320 individual centromere measurements per replicate (40 centromeres signal/cell × 8 cells = 320). Differences in fluorescent intensity between strains were assessed by ANOVA (baseR). Fluorescent intensity is represented in arbitrary units (AU). Thresholding and signal quantification were performed using Fiji [64].

### Evaluating signals of meiotic drive in the diversity outbred mapping population

We utilized genotype probability data from five Diversity Outbred (DO) mapping studies conducted on mouse cohorts from outbreeding generations 11 to 22 (Bult MegaMUGA, Svenson-183 MegaMUGA, Churchill-181 MegaMUGA, Attie-232 GigaMUGA, and Chesler-192 MegaMUGA; all data from https://www.jax.org/research-and-faculty/genetic-diversity-initiative/tools-data/diversity-outbred-database). All DO mice were genotyped at a common set of loci [48]. For mice in each outbreeding generation, we first determined the frequency of each parental haplotype at every genotyped marker. We then looked for linked clusters of markers that exhibit a consistent departure from the expected haplotype frequency (0.125) and that displayed a monotonic increase in the frequency of one or more haplotypes over outbreeding generations.

### Mouse phenotype data

Spearman correlation tests were used to examine relationships between chromosome instability phenotypes and estimated centromere satellite copy number across inbred lab strains. Chromosome instability phenotypes were obtained from the Mills1 dataset deposited in the Mouse Phenome Database [65].

## Supplementary Information

**Additional file 1: Figure S1.** Variation in consensus centromere satellite 15-mers across diverse *Mus musculus*. Boxplots of the distribution of major (yellow) and minor (blue) satellite consensus 15-mer frequencies across inbred strains and wild-caught mouse populations.

**Additional file 2: Figure S2.** Concordance of GC-corrected *k*-mer counts among strains and replicate libraries within a strain. Heatmap of pairwise Pearson correlations between GC-corrected consensus centromere 31-mer frequencies from replicate sequencing libraries across inbred *Mus musculus* strains. Both color intensity and circle size correspond to the magnitude of the R^2^ correlation coefficient. Red lines delimit replicate libraries for single inbred strains.

**Additional file 3: Figure S3**. Consensus centromere 15-mers are the most abundant and the most variable 15-mers in 54 diverse *Mus* genomes. Heatmap displaying the observed frequencies of the 1000 most variable 15-mers (columns) across 54 diverse samples (rows). The color scale represents the normalized frequency of 15-mers. 15-mers present in the *Mus musculus* minor and major satellite consensus sequences are noted by the blue and yellow boxes, respectively.

**Additional file 4: Figure S4.** Inbred strains and wild-caught mice exhibit distinct consensus centromere *k*-mer frequencies. Principal component analysis of (A) major and (B) minor satellite consensus 31-mer frequencies in inbred strains and wild-caught *M. musculus* samples.

**Additional file 5: Figure S5.** Centromere consensus 31-mer estimates of relative copy number strongly correlate with the percentage of reads mapping to the centromere consensus. Correlation plots for the median frequency of GC-corrected centromere consensus 31-mers and the percentage of reads mapping to the centromere consensus for the (A) minor and (B) major satellite. Subspecies are represented by color. Inbred and wild-caught mice are distinguished by shape.

**Additional file 6: Figure S6.** Non-consensus nucleotide proportions at positions 75, 78, and 79 along the minor satellite consensus sequence. The x-axis represents the fraction of centromeric *k*-mers with each nucleotide at the specified position. Each strain is depicted as a single row. The consensus nucleotide at each position is indicated by a black outline.

**Additional file 7: Figure S7.** No correlation between micronuclei frequency and centromere satellite consensus copy number. Spearman correlations between the proportion of peripheral blood cells (red blood cells and micronuclei) with micronuclei and median minor (left) or major (right) satellite 31-mer frequencies. The proportion of cells with micronuclei was determined for 12-month-old mice (top) and 20-month-old mice (bottom).

**Additional file 8: Figure S8.** Haplotype frequencies at centromere-proximal regions in the Diversity Outbred populations are not consistent with strong centromere drive. Chromosome coordinates of genotyped markers in megabases (Mb) are provided on the x-axis. The difference in the frequency of each strain haplotype between generation 22 and generation 11 is shown on the y-axis. Line colors correspond to each of the 8 DO founder strains.

**Additional file 9: Figure S9.** Centromere consensus 31-mer frequencies exhibit low variance between independent sequencing libraries and among wild-caught individuals from a single population. Boxplots reveal the distribution of minor centromere satellite 31-mer frequencies for individual sequencing libraries and wild-caught individuals.

**Additional file 10: Table S1.** Results from TukeyHSD post-hoc tests comparing the distributions of minor and major satellite 31-mer frequencies in inbred strains and wild-caught mice.

**Additional file 11: Table S2.** The frequency of relaxed edit distance centromere satellite 31-mers in the mouse reference (mm10) genome.

**Additional file 12: Table S3.** House mouse (*Mus*) whole genome sequence samples. Numbers represented in the data source column correspond to the following data sources: (1) The Mouse Genomes Project Release 1502/REL-1502 [32]; (2) Whole genome sequencing of *Mus caroli* and *Mus pahari* [31]; (3) Wild Mouse Genomes Project [33]. *We excluded Mouse Genomes Project libraries with read length < 75 bp.

**Additional file 13: Table S4.** Sequences and primers used for FISH experiments.

## Data Availability

Whole genome sequences analyzed for inbred mouse strains are available as bam files on the Sanger Mouse Genomes project page: https://www.sanger.ac.uk/data/mouse-genomes-project/. Whole genome sequences analyzed for wild mouse genomes are available as bam files on the Wild Mouse Genomes project ftp site (http://wwwuser.gwdg.de/~evolbio/evolgen/wildmouse/) and as fastq files on the Short Read Archive under Project Numbers PRJEB9450, PRJEB11742, PRJEB14167, and PRJEB2176. Whole genome sequences analyzed for *M. caroli* and *M. pahari* are available as fastq files on the Short Read Archive under Project Numbers PRJEB14895 and PRJEB14896 respectively. All scripts used to generate and analyze data in this study are publicly available through github (https://github.com/umaarora/KmerDigging).

## Abbreviations

qFISH: Quantitative fluorescence in situ hybridization
ANOVA: Analysis of Variance
PCA: Principal Component Analysis
CDI: Centromere Diversity Index
CENP-B: Centromere protein B
CENP-A: Centromere protein A
DO: Diversity Outbred
Mb: Megabases
bam: Binary alignment map
FIJI: Fiji Is Just ImageJ
