# Supplementary figures and images for: Population and subspecies diversity at mouse centromere satellites
Source: BMC Genomics. 2021 Apr 17;22:279. doi: 10.1186/s12864-021-07591-5 (PMC8052823; doi:10.1186/s12864-021-07591-5)

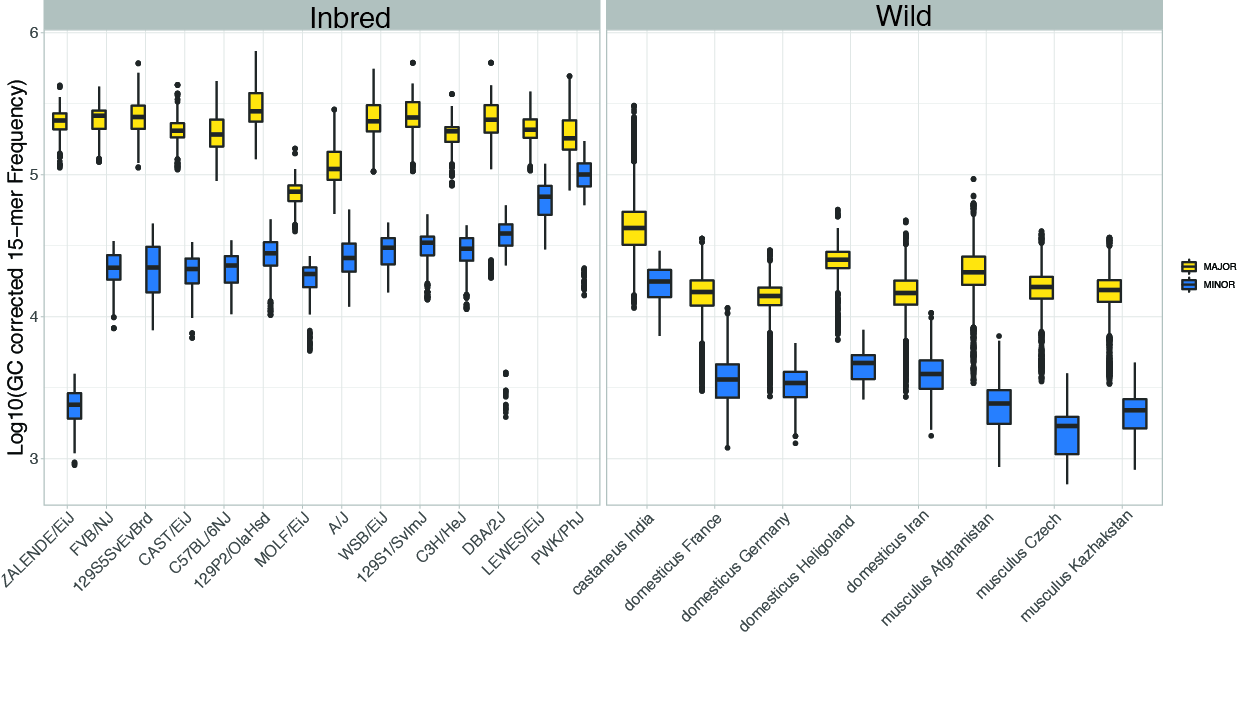

Supplement: Supplementary file 1 — Additional file 1: Figure S1. Variation in consensus centromere satellite 15-mers across diverse Mus musculus. Boxplots of the distribution of major (yellow) and minor (blue) satellite consensus 15-mer frequencies across inbred strains and wild-caught mouse populations. [file 12864_2021_7591_MOESM1_ESM.tif]

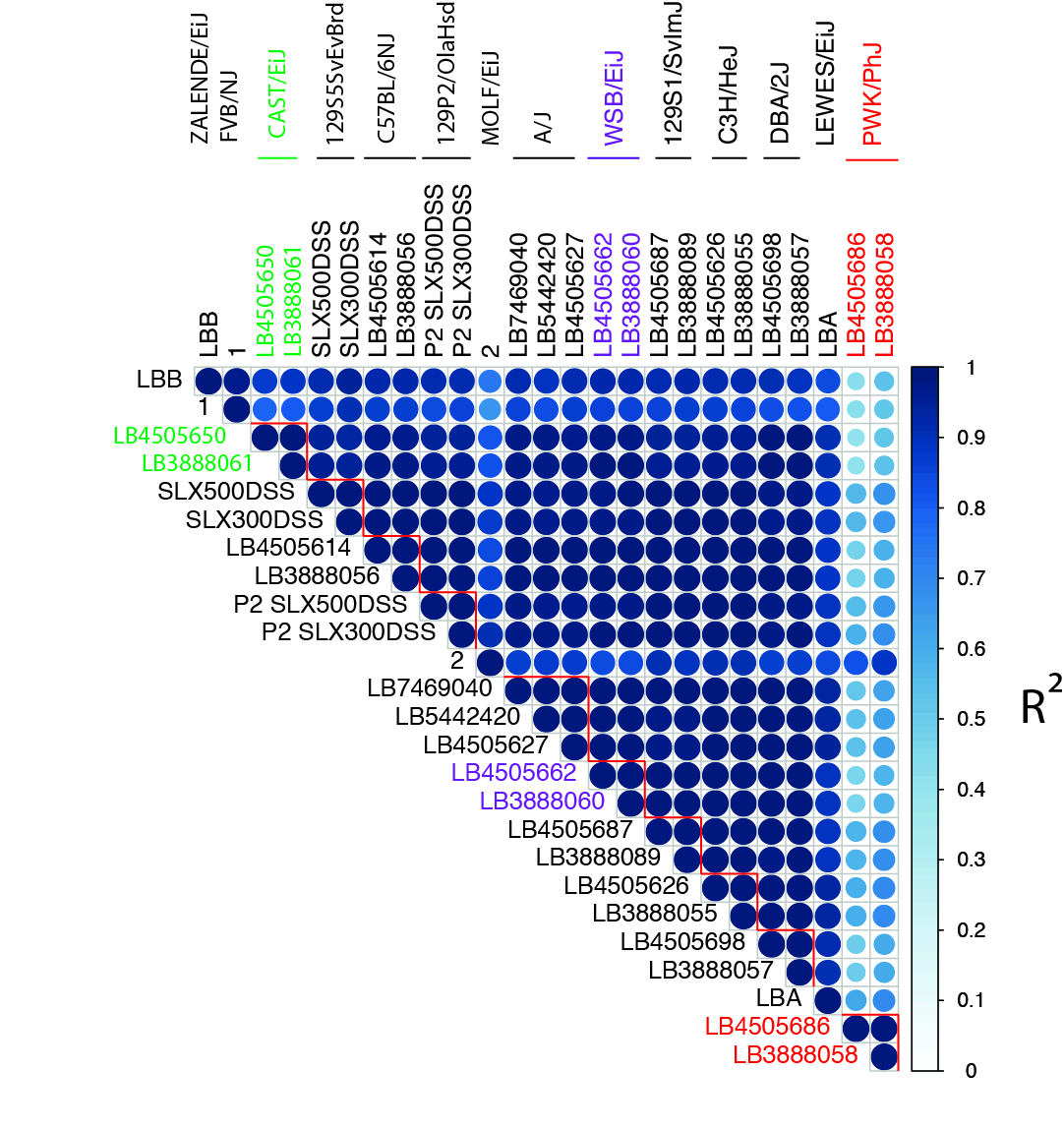

Supplement: Supplementary file 2 — Additional file 2: Figure S2. Concordance of GC-corrected k-mer counts among strains and replicate libraries within a strain. Heatmap of pairwise Pearson correlations between GC-corrected consensus centromere 31-mer frequencies from replicate sequencing libraries across inbred Mus musculus strains. Both color intensity and circle size correspond to the magnitude of the R2 correlation coefficient. Red lines delimit replicate libraries for single inbred strains. [file 12864_2021_7591_MOESM2_ESM.tif]

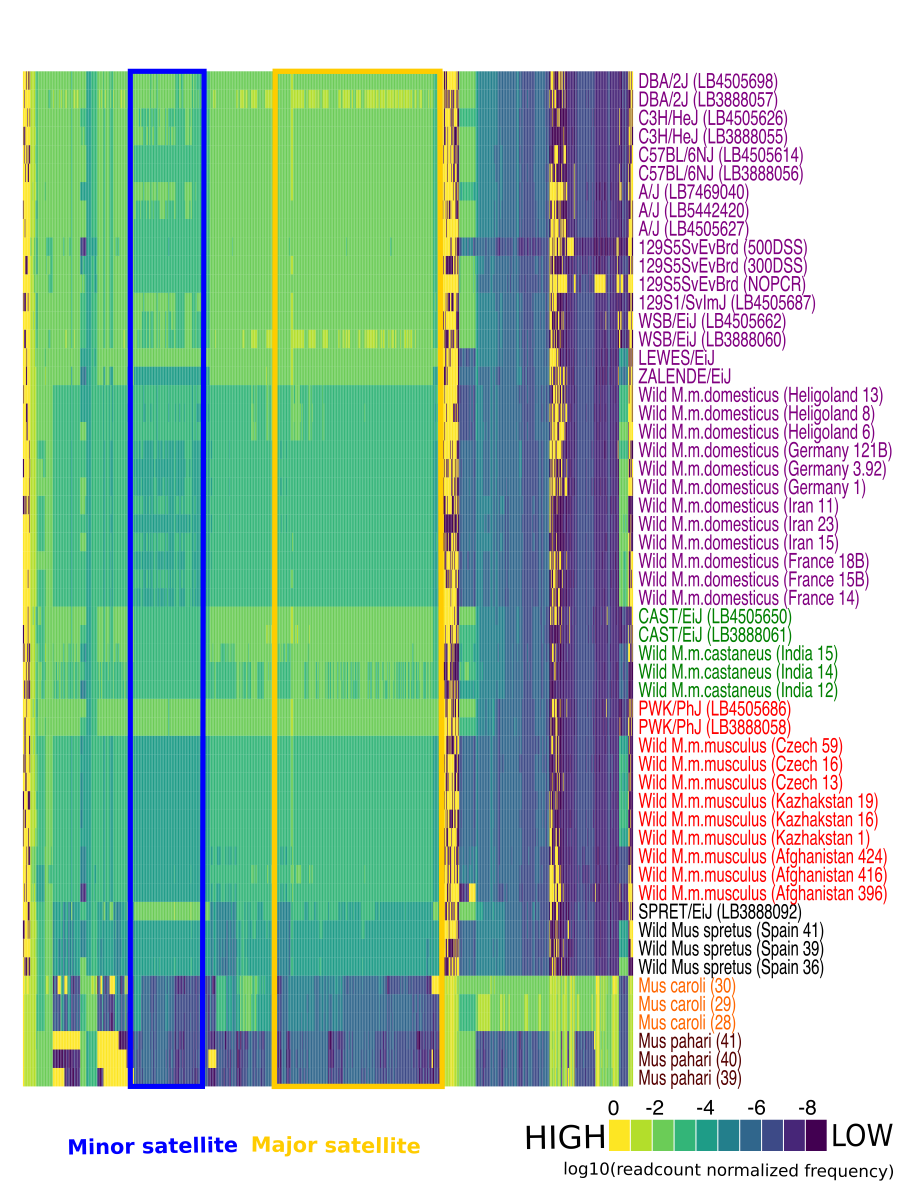

Supplement: Supplementary file 3 — Additional file 3: Figure S3. Consensus centromere 15-mers are the most abundant and the most variable 15-mers in 54 diverse Mus genomes. Heatmap displaying the observed frequencies of the 1000 most variable 15-mers (columns) across 54 diverse samples (rows). The color scale represents the normalized frequency of 15-mers. 15-mers present in the Mus musculus minor and major satellite consensus sequences are noted by the blue and yellow boxes, respectively. [file 12864_2021_7591_MOESM3_ESM.tif]

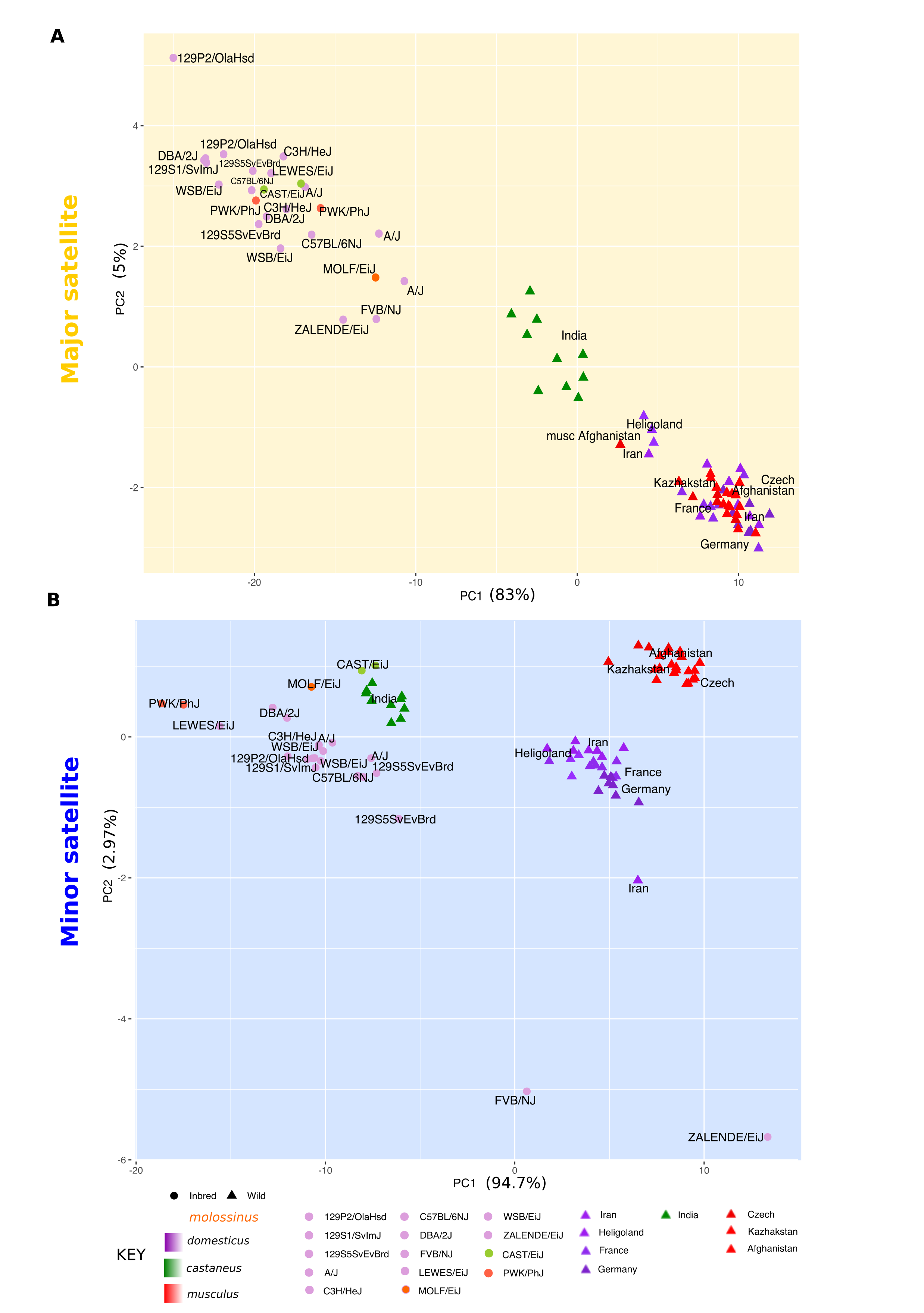

Supplement: Supplementary file 4 — Additional file 4: Figure S4. Inbred strains and wild-caught mice exhibit distinct consensus centromere k-mer frequencies. Principal component analysis of (A) major and (B) minor satellite consensus 31-mer frequencies in inbred strains and wild-caught M. musculus samples. [file 12864_2021_7591_MOESM4_ESM.png]

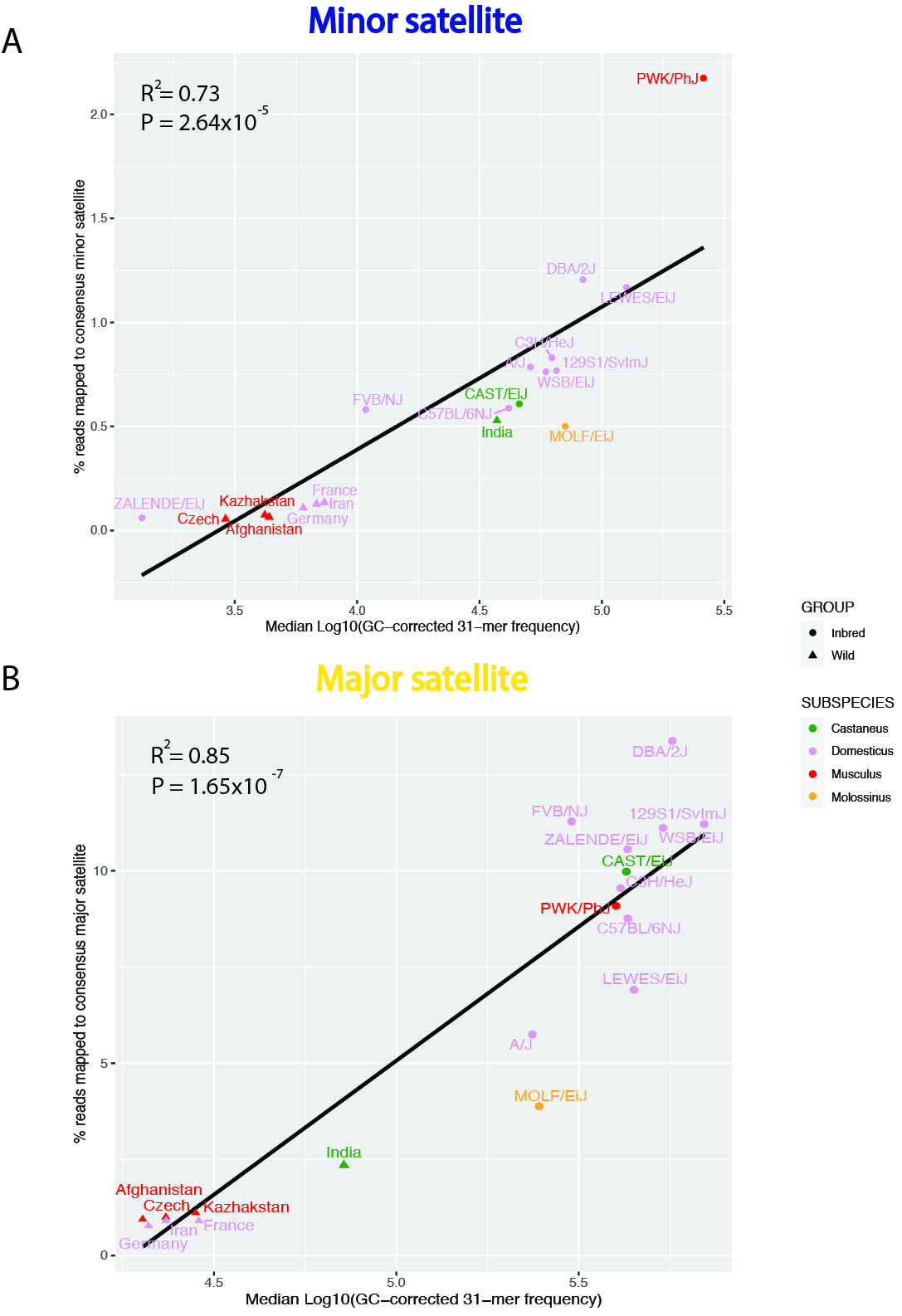

Supplement: Supplementary file 5 — Additional file 5: Figure S5. Centromere consensus 31-mer estimates of relative copy number strongly correlate with the percentage of reads mapping to the centromere consensus. Correlation plots for the median frequency of GC-corrected centromere consensus 31-mers and the percentage of reads mapping to the centromere consensus for the (A) minor and (B) major satellite. Subspecies are represented by color. Inbred and wild-caught mice are distinguished by shape. [file 12864_2021_7591_MOESM5_ESM.tif]

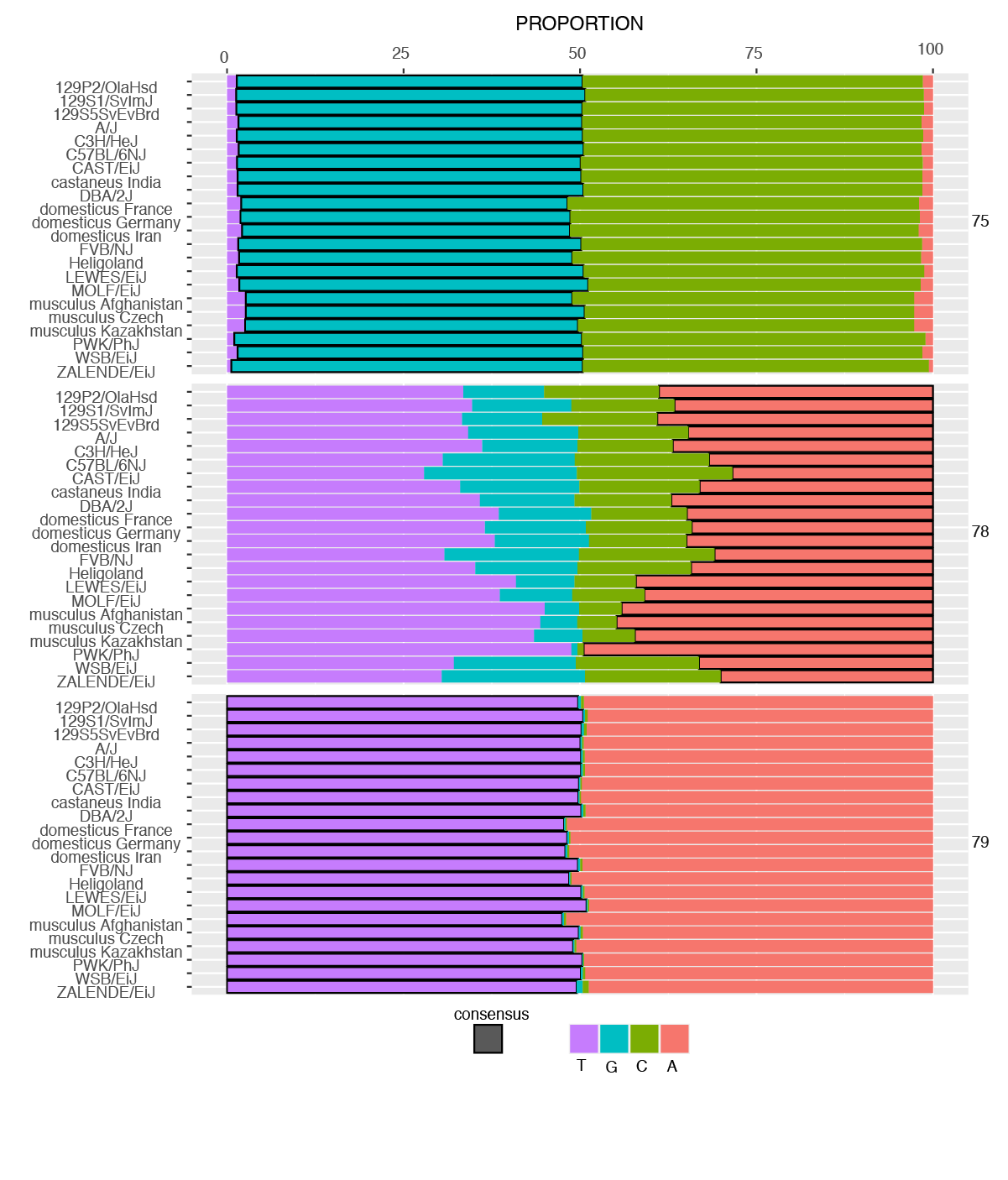

Supplement: Supplementary file 6 — Additional file 6: Figure S6. Non-consensus nucleotide proportions at positions 75, 78, and 79 along the minor satellite consensus sequence. The x-axis represents the fraction of centromeric k-mers with each nucleotide at the specified position. Each strain is depicted as a single row. The consensus nucleotide at each position is indicated by a black outline. [file 12864_2021_7591_MOESM6_ESM.tif]

proportion of cells with micronuclei

M12

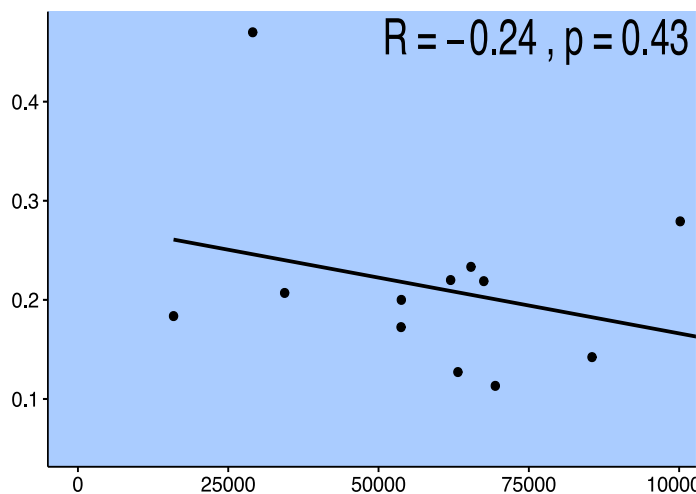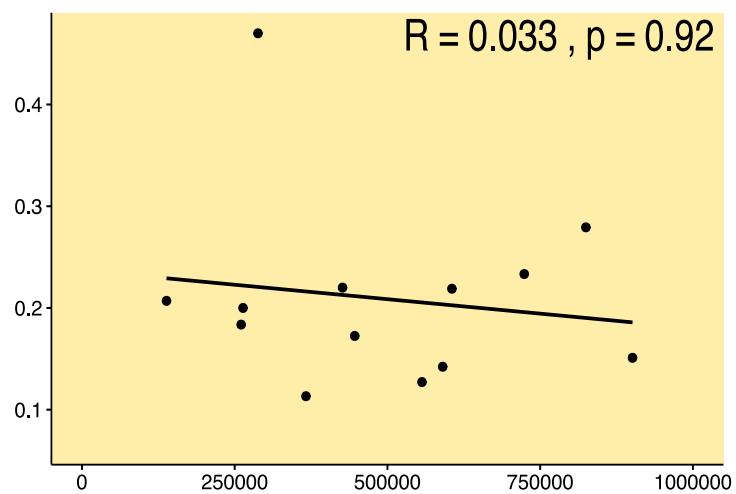

M20

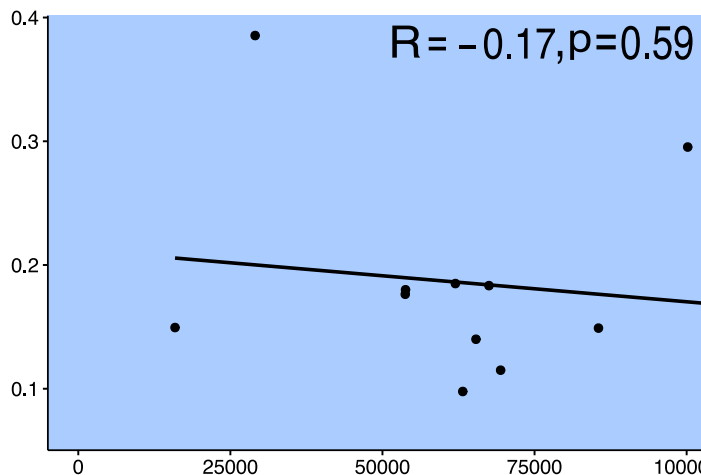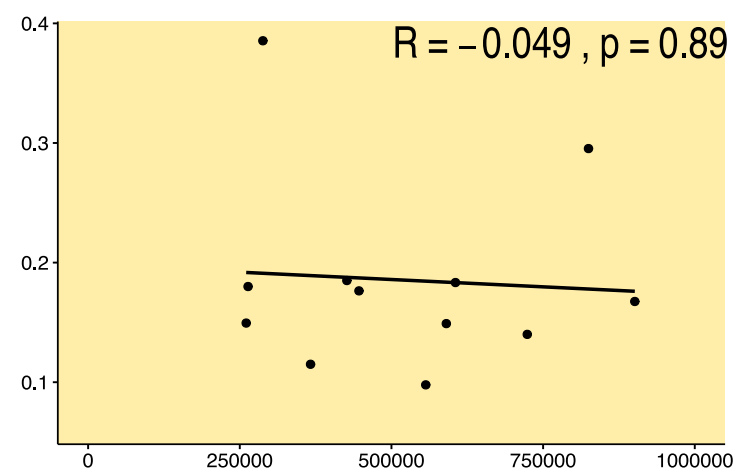

Consensus centromere satellite copy number

Supplement: Supplementary file 7 — Additional file 7: Figure S7. No correlation between micronuclei frequency and centromere satellite consensus copy number. Spearman correlations between the proportion of peripheral blood cells (red blood cells and micronuclei) with micronuclei and median minor (left) or major (right) satellite 31-mer frequencies. The proportion of cells with micronuclei was determined for 12-month-old mice (top) and 20-month-old mice (bottom). [file 12864_2021_7591_MOESM7_ESM.pdf]

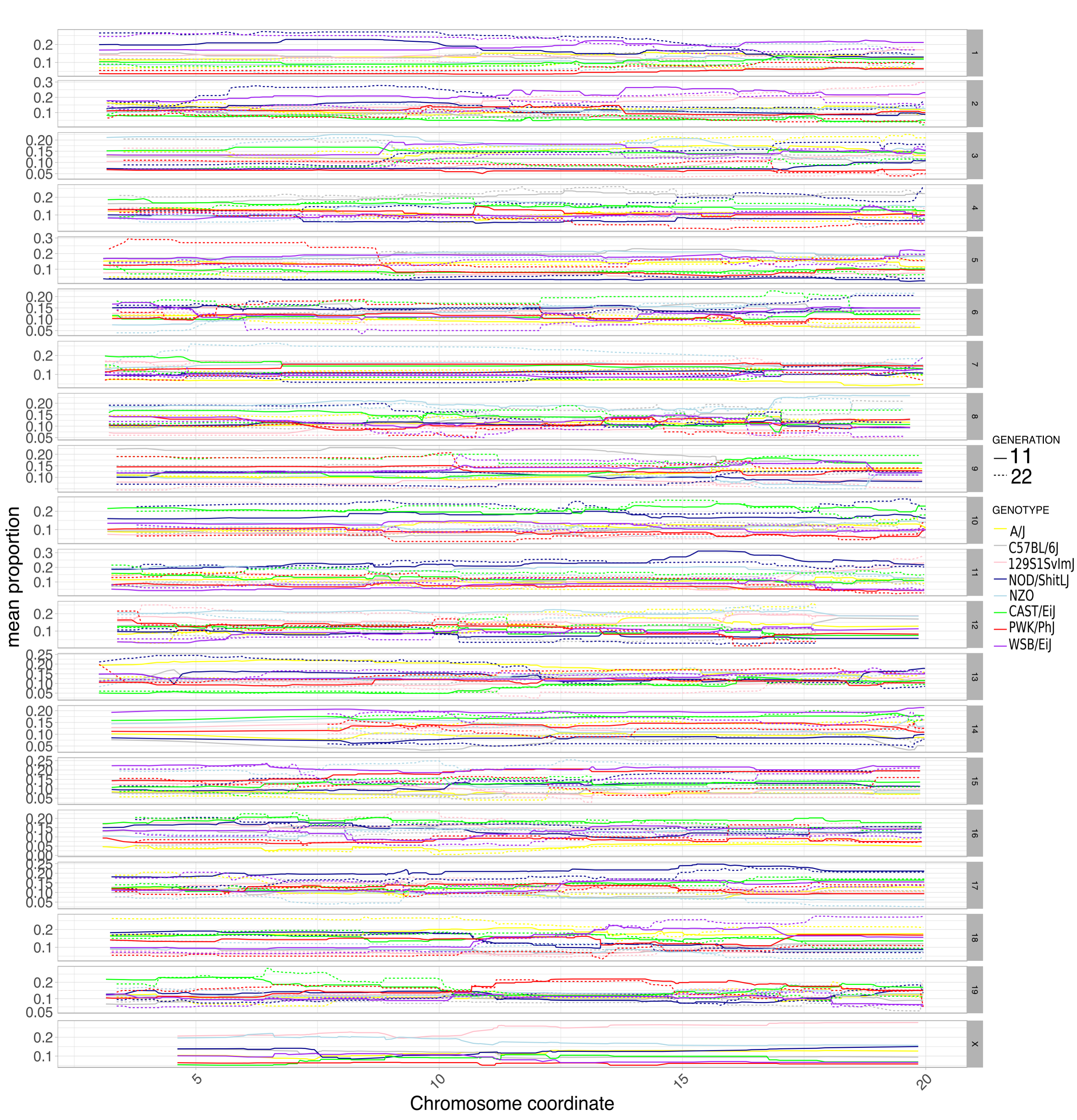

Supplement: Supplementary file 8 — Additional file 8: Figure S8. Haplotype frequencies at centromere-proximal regions in the Diversity Outbred populations are not consistent with strong centromere drive. Chromosome coordinates of genotyped markers in megabases (Mb) are provided on the x-axis. The difference in the frequency of each strain haplotype between generation 22 and generation 11 is shown on the y-axis. Line colors correspond to each of the 8 DO founder strains. [file 12864_2021_7591_MOESM8_ESM.pdf]

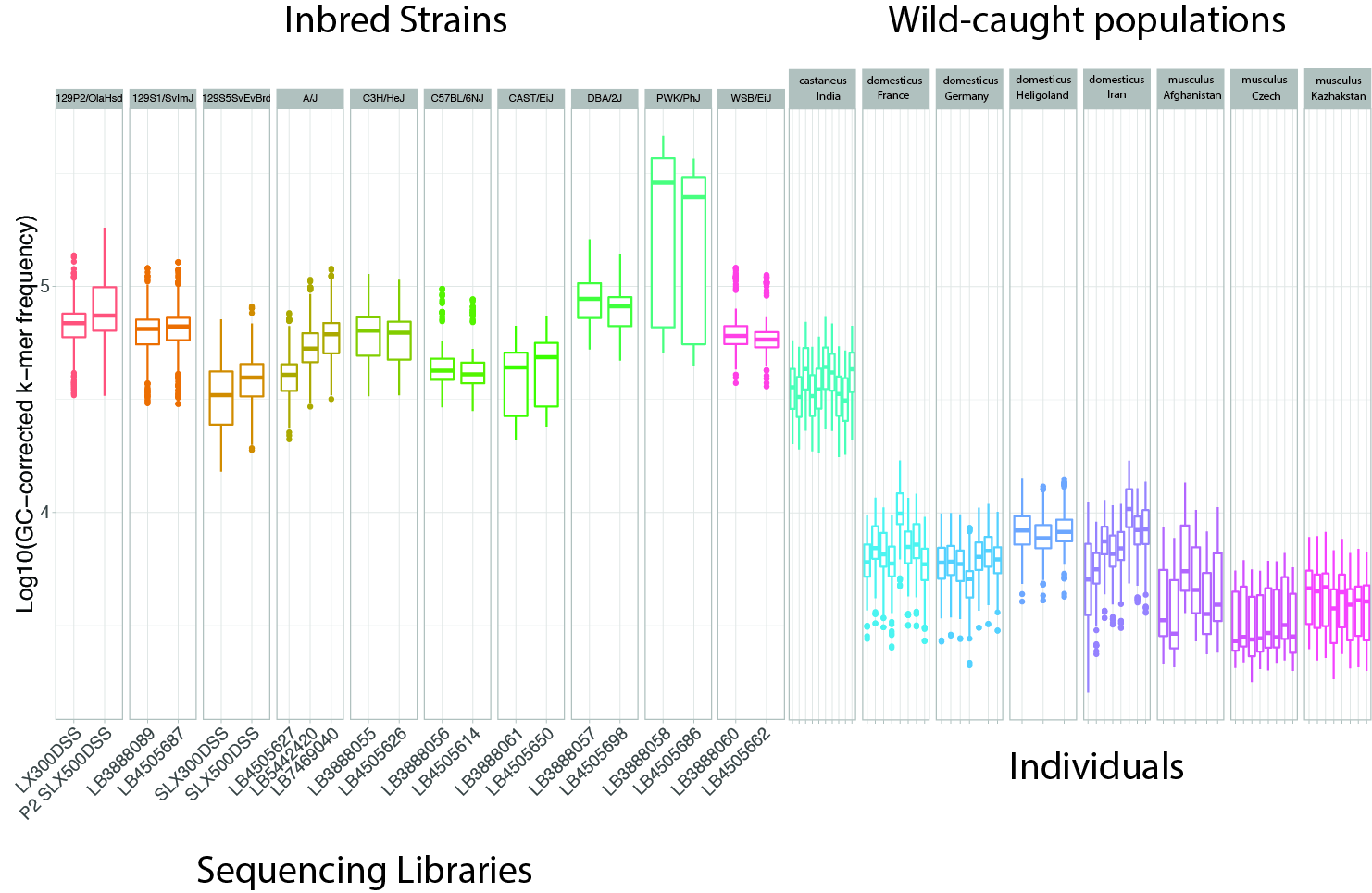

Supplement: Supplementary file 9 — Additional file 9: Figure S9. Centromere consensus 31-mer frequencies exhibit low variance between independent sequencing libraries and among wild-caught individuals from a single population. Boxplots reveal the distribution of minor centromere satellite 31-mer frequencies for individual sequencing libraries and wild-caught individuals. [file 12864_2021_7591_MOESM9_ESM.tif]
